# Interspecific and host-related gene expression patterns in nematode-trapping fungi

**DOI:** 10.1186/1471-2164-15-968

**Published:** 2014-11-11

**Authors:** Karl-Magnus Andersson, Dharmendra Kumar, Johan Bentzer, Eva Friman, Dag Ahrén, Anders Tunlid

**Affiliations:** Department of Biology, Microbial Ecology Group, Lund University, Ecology Building, 223 62 Lund, Sweden; Department of Genetics and Plant Breeding, College of Agriculture, Narendra Deva University of Agriculture and Technology, Kumarganj, Faizabad, 224229 Uttar Pradesh (U.P.) India; Department of Biology, BILS Bioinformatics Infrastructure for Life Sciences, Lund University, Ecology Building, 223 62 Lund, Sweden

**Keywords:** Comparative transcriptomics, *Heterodera schachtii*, *Meloidogyne hapla*, Nematode-trapping fungi

## Abstract

**Background:**

Nematode-trapping fungi are soil-living fungi that capture and kill nematodes using special hyphal structures called traps. They display a large diversity of trapping mechanisms and differ in their host preferences. To provide insights into the genetic basis for this variation, we compared the transcriptome expressed by three species of nematode-trapping fungi (*Arthrobotrys oligospora*, *Monacrosporium cionopagum* and *Arthrobotrys dactyloides*, which use adhesive nets, adhesive branches or constricting rings, respectively, to trap nematodes) during infection of two different plant-pathogenic nematode hosts (the root knot nematode *Meloidogyne hapla* and the sugar beet cyst nematode *Heterodera schachtii*).

**Results:**

The divergence in gene expression between the fungi was significantly larger than that related to the nematode species being infected. Transcripts predicted to encode secreted proteins and proteins with unknown function (orphans) were overrepresented among the highly expressed transcripts in all fungi. Genes that were highly expressed in all fungi encoded endopeptidases, such as subtilisins and aspartic proteases; cell-surface proteins containing the carbohydrate-binding domain WSC; stress response proteins; membrane transporters; transcription factors; and transcripts containing the Ricin-B lectin domain. Differentially expressed transcripts among the fungal species encoded various lectins, such as the fungal fruit-body lectin and the D-mannose binding lectin; transcription factors; cell-signaling components; proteins containing a WSC domain; and proteins containing a DUF3129 domain. A small set of transcripts were differentially expressed in infections of different host nematodes, including peptidases, WSC domain proteins, tyrosinases, and small secreted proteins with unknown function.

**Conclusions:**

This is the first study on the variation of infection-related gene expression patterns in nematode-trapping fungi infecting different host species. A better understanding of these patterns will facilitate the improvements of these fungi in biological control programs, by providing molecular markers for screening programs and candidates for genetic manipulations of virulence and host preferences.

**Electronic supplementary material:**

The online version of this article (doi:10.1186/1471-2164-15-968) contains supplementary material, which is available to authorized users.

## Background

Soil contains a diverse range of fungi that are parasites on nematodes [1]. These fungi include the nematode-trapping fungi, which have specific hyphal structures in which the nematodes can be trapped. The interest in studying these fungi is due to their potential use as biological control agents for plant and animal parasitic nematodes [2]. The traps of the nematode-trapping fungi develop from hyphae and can be formed spontaneously or be induced in response to signals from the environment [3]. There is a large variation in the morphology of the traps, and the type of trap depends on the species [4]. In some species, the traps consist of an erect branch that is covered by an adhesive material. In other species such as in the well-studied *Arthrobotrys oligospora*, the trap is a three-dimensional net. A third type of trap is the adhesive knob, which is a single celled structure. Finally, there are some species that capture nematodes using a mechanical trap called a constricting ring [4]. Despite the large morphological variation in trapping structures, phylogenetic analyses inferred from molecular data has shown that the majority of the nematode-trapping fungi belong to a monophyletic group consisting of a single family of the order Orbiliales (Ascomycota) [5–8]. Furthermore, these studies have shown that the nematode-trapping fungi have evolved along two major lineages: one basal lineage leading to species with constricting rings and one lineage containing species that form adhesive traps, including three-dimensional networks, knobs and branches [5–8].

The trapping mechanisms of the species with constricting rings and those with adhesive traps are distinctively different [3]. The constricting ring consists of three cells. When the nematode enters the ring, the cells inflate and the nematode is trapped. The closure is very rapid (0.1 s) and is triggered by pressure of the nematode on the constricting-ring cells [9]. Ultrastructural examinations revealed that the cell wall of the constricting-ring cells is folded; when the cells inflate, the folded cell wall balloons out and forms the new cell wall [10, 11]. The adhesive trap is surrounded by a layer of fibrillar, extracellular polymers. Although the molecular mechanism has not yet been characterized, ultrastructural studies have shown that the fibrillar layer is reorganized during the attachment of the traps to the nematode cuticle [12]. Following the trapping of nematodes, the infection mechanisms appear to be rather similar in the species with constricting rings and adhesive traps: the fungus forms a penetration tube that pierces the nematode cuticle. During penetration the nematode becomes paralyzed. Subsequently, the internal tissues are rapidly colonized and digested by fungal hyphae [13].

In laboratory assays, most nematode-trapping fungi can trap and infect a range of different nematode species [14, 15]. However, there are a number of studies showing that different species and even strains of nematode-trapping fungi can vary in their host preferences. For example, *in vitro* predacity tests of four nematode-trapping fungi showed that the constricting-ring species *Arthrobotrys dactyloides* was the most efficient species in capturing and killing the root knot nematode *Meloidogyne graminicola* [16]. Further studies on *A. dactyloides* showed that even strains of this species differed in their predacity to *Meloidogyne incognita* [17]. Significant differences in the susceptibility to nematode-trapping fungi have also been shown in field trials with the cyst nematode *Heterodera schachtii* and the root knot nematode *Meloidogyne javanica* [18].

Recently, the infection mechanism of nematode-trapping fungi has been examined using the tools of genomics, transcriptomics and proteomics. The genomes of two nematode-trapping fungi have been published; the net-forming *Arthrobotrys oligospora* [19] and the adhesive knob-forming *Monacrosporium haptotylum* [20]. The two genomes are similar in size and consist of ~62% core genes that are shared with other fungi, ~20% genes that are specific for the two species and ~16% genes that are unique for each genome [20]. Comparative genome analysis showed that the genomes of nematode-trapping fungi have been expanded in a number of gene families, including extracellular peptidases such subtilisins; homologs to several virulence factors identified in plant-pathogenic fungi; and families of putative cell-surface adhesins containing carbohydrate-binding domains such as the WSC domain and the mucin domain [20]. Transcriptome analysis showed that *M. haptotylum* expresses a unique set of genes during the early stages of infection of the nematode *Caenorhabditis briggsae*. Among these is a large proportion that belongs to gene families that are significantly expanded in the nematode-trapping fungi. Transcripts encoding small secreted proteins (SSPs) and many species-specific genes were also highly expressed during the early phase of infection. Many of them were orphans, that is, genes lacking both homologs and Pfam domains [20, 21]. Furthermore, quantitative proteomics revealed proteins that were significant upregulated in the knob compared with the vegetative mycelia in *M. haptotylum*. Among the upregulated proteins were peptidases, tyrosinase and proteins containing the WSC domain [22].

In this study, we have examined in more detail the molecular basis of the infection process in nematode-trapping fungi that have various trapping mechanisms, including adhesive nets (*A. oligospora*), adhesive branches (*Monacrosporium cionopagum*) and constricting rings (*A. dactyloides*) [4, 13]. Two plant-parasitic nematodes were used as hosts, the root knot nematode *Meloidogyne hapla* and the sugar beet cyst nematode *H. schachtii*. These are both sedentary endoparasites belonging to the group of nematodes that causes the most damages to crops [23]. Comparative transcriptome analysis of the infection, including the adhesion, penetration and digestion stages, showed that the divergence in interspecific gene expression was significantly larger than that related to the nematode host used. We identified a common set of genes that were expressed by all three fungi and a more variable set that were regulated depending on either the fungal species or the nematode host.

## Results and discussion

### Infection experiments

**Figure 1:**
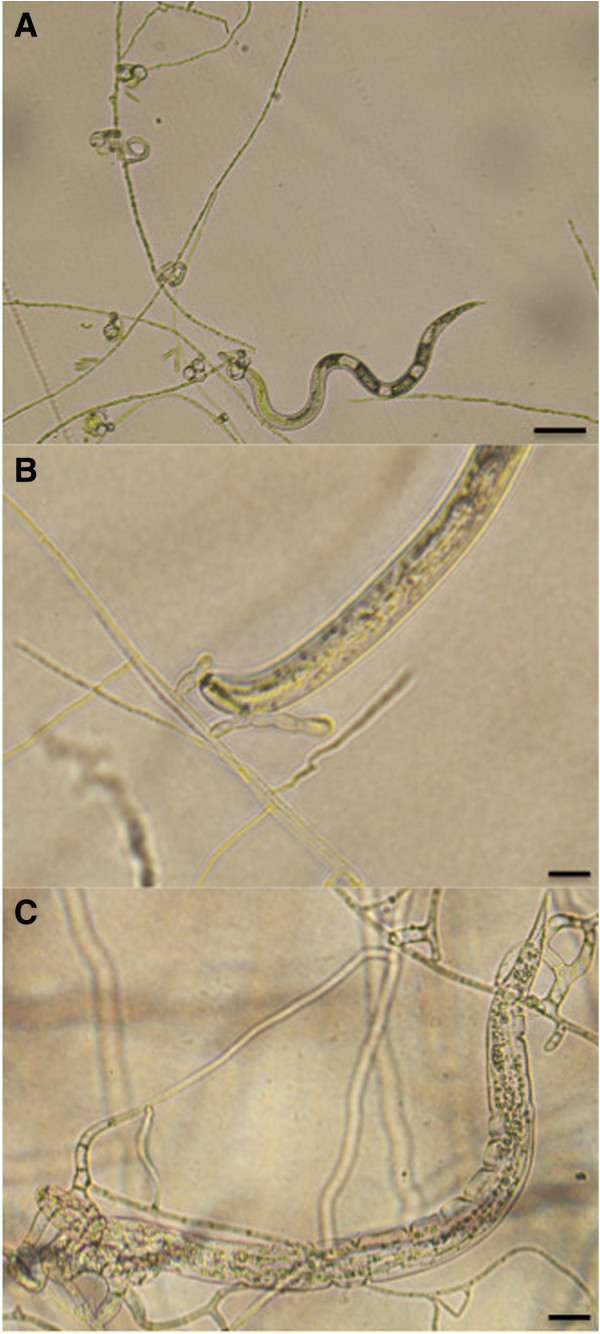
**Micrographs of fungal and nematode interactions. (A)** *Arthrobotrys dactyloides* (constricting rings) after trapping the root knot nematode *Meloidogyne hapla* (bar = 50 μm). **(B)** *Monacrosporium cionopagum* (adhesive branches) after trapping and immobilizing the sugar beet cyst nematode *Heterodera schachtii* (bar = 20 μm). **(C)** *Arthrobotrys oligospora* (adhesive nets) after trapping, immobilization and colonization of *H. schachtii* (bar = 20 μm).

The nematodes *M. hapla* or *H. schachtii* were added to plates containing the nematode-trapping fungi *A. oligospora*, *M. cionopagum* or *A. dactyloides* and the infection was followed under a light microscope (Figure 1). The following five combinations of fungi and nematodes were examined: *A. oligospora* and *M. hapla* (designated Ao(Mh)), *A. oligospora* and *H. schachtii* (Ao(Hs)), *A. dactyloides* and *M. hapla* (Ad(Mh)), *A. dactyloides* and *H. schachtii* (Ad(Hs)), and *M. cionopagum* and *H. schachtii* (Mc(Hs)). There was a large difference in the infection rate, both between the nematodes and between the fungi (Table 1). Cyst nematodes (*H. schachtii)* showed lower susceptibility to nematode-trapping fungi than did root knot nematodes (*M. hapla*), which is in agreement with an earlier study by Jaffee and Muldoon [18]. This might be due to differences in the composition of the nematode cuticle [24]. Between the fungi, *A. dactyloides* infected *M. hapla* at a faster rate than *A. oligospora* infected *M. hapla. A. dactyloides* colonized *H. schachtii* at a faster rate than the other species, especially *M. cionopagum*. The observation that species with constricting rings are more efficient in capturing and killing nematodes than nematode-trapping fungi with other types of trapping structures has been made in earlier studies [16].

**Table 1:** **Infection of plant parasitic nematodes by nematode-trapping fungi** ^**a**^

	Ad(Mh)	Ao(Mh)	Ad(Hs)	Ao(Hs)	Mc(Hs)
**Trapped**					
3 h	80.3 (3.8)				
7 h		40.5 (2.6)			
12 h			30.3 (3.7)	50.0 (3.3)	50.6 (2.5)
**Paralyzed**					
12 h	90.8 (2.5)	30.4 (2.4)			
24 h			31.0 (2.4)		
32 h				50.7 (2.9)	
36 h					60.7 (4.1)
**Colonized**					
20 h	92.7 (3.2)				
24 h		64.7 (2.3)			
36 h			35.3 (3.0)		
40 h				60.3 (2.7)	
48 h					55.2 (2.4)

^a^Shown is the percentage (mean (SD, n = 10)) of the added nematodes that were trapped, paralyzed and colonized. Ao(Mh) denotes *A. oligospora* infecting *M. hapla*; Ao(Hs), *A. oligospora* infecting *H. schachtii*; Ad(Mh), *A. dactyloides* infecting *M. hapla*; Ad(Hs), *A. dactyloides* infecting *H. schachtii*; and Mc(Hs), *M. cionopagum* infecting *H. schachtii*.

### Characterization of the transcriptome libraries

**Figure 2:**
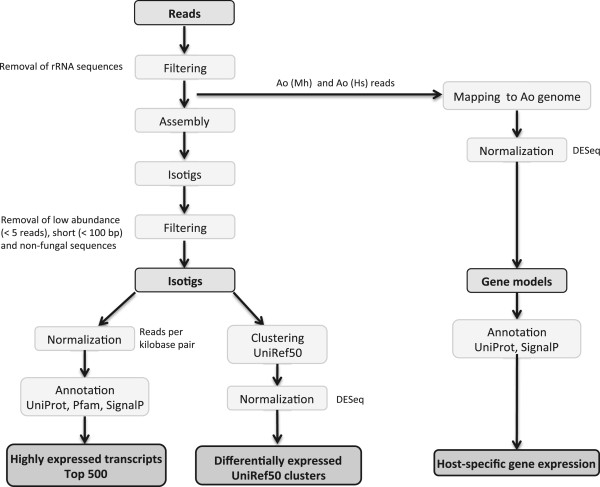
**Flowchart of the data analysis.** Five different cDNA libraries were sequenced and the reads were used to generate three different data sets. First, the data set “Highly expressed transcripts” were retrieved by assembling the reads of each library into isotigs (ranscripts) and normalizing the read counts. Using this approach, the 500 most highly expressed transcripts in each library were retrieved (the “Top 500” data set). Second, the data set “Differentially expressed UniRef50 clusters” was obtained by matching the isotig sequences using BLASTX [25] to UniRef50 clusters [26]. The procedure organized the isotigs into putative orthologs for which expression levels could directly be compared between the five libraries. Third, to identify the data set “Host-specific gene expression” the reads from the two libraries of *A. oligospora* were mapped to the genome sequence of this fungus [19]. Ao(Mh) denotes *A. oligospora* and *M. hapla*; Ao(Hs), *A. oligospora* and *H. schachtii*; Ad(Mh), *A. dactyloides* and *M. hapla*; Ad(Hs), *A. dactyloides* and *H. schachtii*; and Mc(Hs), *M. cionopagum* and *H. schachtii*. Further details of the libraries are shown in Table 2.

The number of reads obtained by the 454 sequencing of the five cDNA libraries corresponding to these species combinations ranged from 70 061 to 245 264 (Table 2). Based on these sequences, three different data sets were created (Figure 2). To generate the “Highly expressed transcripts” data set, the reads were assembled into isotigs (transcripts), and low abundance reads (<5 reads), short isotigs (<100 base pairs, bp) and non-fungal sequences were removed. The number of the filtered isotigs in the five libraries varied between 1 318 and 5 140 and their average sizes varied between 1 008 to 1 237 bp. Almost all of the assembled isotigs (98.9%) had fungal matches: a few matched species from Nematoda (0.9%) and a few matched other species (0.2%). To identify the set of transcripts that was most highly expressed in each library, the reads of the isotigs were normalized using the reads per kilobase pair method. This method fitted better than the reads per kilobase per million read (RPKM) method to the expectation that most transcripts have similar relative expression abundance between samples (Additional file 1). In each library, the 500 most expressed isotigs (the “Top 500 transcripts”) were analyzed.

**Table 2:** **Characterization of the transcriptome libraries**

Library ^a^	Ao(Mh)	Ao(Hs)	Ad(Mh)	Ad(Hs)	Mc(Hs)
*Reads*					
Total number of reads	114 418	70 061	226 301	191 632	245 264
Filtered reads^b^	97 770	58 208	183 433	141 506	206 854
*Isotigs*					
Total number of isotigs	2 663	1 354	4 517	4 003	5 258
Fungal isotigs	2 514	1 261	4 021	3 560	4 741
Nematode isotigs	11	20	21	18	44
Others	0	5	8	4	16
Filtered isotigs^c^	2 634	1 318	4 428	3 926	5 140
(Average size, bp)	(1 160)	(1 008)	(1 237)	(1 128)	(1 167)
Isotigs with Pfam	1 730	856	2 317	2 504	3 071
Number of unique Pfam	1 251	707	1 555	1 592	1 847
Isotigs with UniRef50	2 485	1 239	3 916	3 472	4 601
Unique UniRef50	2 326	1 174	3 101	3 230	3 981
*Mapping to Ao genome*					
Number of mapped reads	73 623	40 051			
Number of gene models^d^	7 351	6 377			

^a^The following five combinations of fungi and nematodes were characterized: *A. oligospora* and *M. hapla* (Ao(Mh)), *A. oligospora* and *H. schachtii* (Ao(Hs)), *A. dactyloides* and *M. hapla* (Ad(Mh)), *A. dactyloides* and *H. schachtii* (Ad(Hs)), and *M. cionopagum* and *H. schachtii* (Mc(Hs)).

^b^Number of reads after removal of rRNA sequences.

^c^Number of isotigs after removal of non-fungal sequences, low abundance isotigs (less than 5 reads), and short isotigs (<100 bp).

^d^Number of gene models that have ≥1 read.

Comparing transcriptomes between species with not yet sequenced genomes is challenging due to the difficulties in identifying one-to-one orthologs. To circumvent this problem, we here clustered the isotigs into UniRef50 clusters. UniRef50 clusters are based on pre-computed sequence clusters of the UniProt database that have at least 50 percent similarity and 80 percent coverage [26]. Istotigs were only grouped to UniRef50 clusters if passing a given threshold value (1e-10) and only one isotig per fungal species (displaying the highest sequence similarity) were assigned to a given UniRef50 cluster. A recent study including data from seven fungal genomes revealed that the grouping of gene sequences into UniRef50 clusters using the described procedure are in close agreement with traditional ortholog clustering methods (Canbäck *et al.,* manuscript in preparation). Furthermore, the risk of clustering non-orthologous gene duplicates into a given UniRef50 cluster is reduced in nematode-trapping due to the rapid divergence of gene duplicates generated by repeat induced point (RIP) mutations [19, 20]. In our analyses, 15 713 of the in total 17 446 isotigs matched to 6 520 unique UniRef50 protein clusters. The reads of these putative orthologs were normalized with DESeq [27]. Based on the hypothesis that most transcripts are not differentially expressed, the analysis showed that a proper normalization was obtained using the 5% most highly expressed UniRef50 clusters (Additional file 2). This cohort (“Differentially expressed UniRef50 clusters”) contained 326 unique UniRef50 clusters.

The third data set, “Host-specific gene expression”, was generated for identifying genes that were differentially expressed due to the nematode host species. Because the genome of *A. oligospora* is available [19], the analysis focused on comparing the transcriptional response of this fungus when infecting *M. hapla* and *H. schachtii*. The reads from the Ao(Mh) library were mapped to 7 351 genes and those from the Ao(Hs) library to 6 377 genes (Table 2).

### Divergence in gene expression

To compare the functional groups of genes that were expressed in the different libraries, the abundances and expression levels of Pfam domains in the “Top 500 transcripts” data set were analyzed. The number of Pfam domains found in the five libraries varied between 330 and 412 (Additional files 3 and 4). In total, 700 Pfam domains were found in at least one of the libraries.

**Figure 3:**
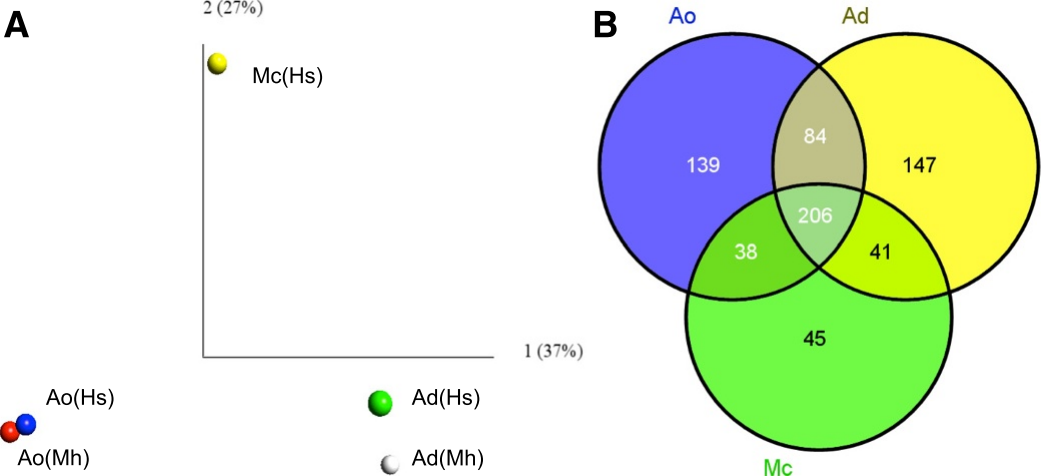
**Expression patterns of Pfam domains among the most highly expressed transcripts. (A)** Principal component analysis (PCA) of the abundance of Pfam domains. Each point in the PCA plot corresponds to a cDNA library. Ao(Mh) denotes *A. oligospora* and *M. hapla*; Ao(Hs), *A. oligospora* and *H. schachtii*; Ad(Mh), *A. dactyloides* and *M. hapla*; Ad(Hs), *A. dactyloides* and *H. schachtii*; and Mc(Hs), *M. cionopagum* and *H. schachtii*. The PCA was performed on the read counts of 700 Pfam domains (log_2_ transformed (counts + 1)). **(B)** Venn diagram of highly expressed Pfam domains. Shown is the distribution of the in total 700 unique Pfam domains that were found among the top 500 most expressed transcripts in *A. oligospora* (Ao), *A. dactyloides* (Ad) and *M. cionopagum* (Mc). ‘Ao’ contains all domains that were found among the 500 most expressed transcript in one or in both of the Ao(Mh) and Ao(Hs) libraries. ‘Ad’ contains all domains that were found among the 500 most expressed transcripts in one or in both of the Ad(Mh) and Ad(Hs) libraries. ‘Mc’ contains all domains that were found among the 500 most expressed transcript in Mc(Hs).

A principal component analysis (PCA) of the abundances of the Pfam domains showed that the libraries mainly clustered according to the fungal species (Figure 3A). The first axis (explaining 37% of the variability) separated the two libraries of *A. dactyloides* (Ad(Mh) and Ad(Hs)) from those of *A. oligospora* (Ao(Mh) and Ao(Hs)). The second axis (27%) separated *M. cionopagum* (Mc(Hs)) from the two *Arthrobotrys* species. Clearly, in both *A. dactyloides* and *A. oligospora*, the divergence in Pfam expression patterns associated with fungal species was larger than that related to the host nematode species.

**Figure 4:**
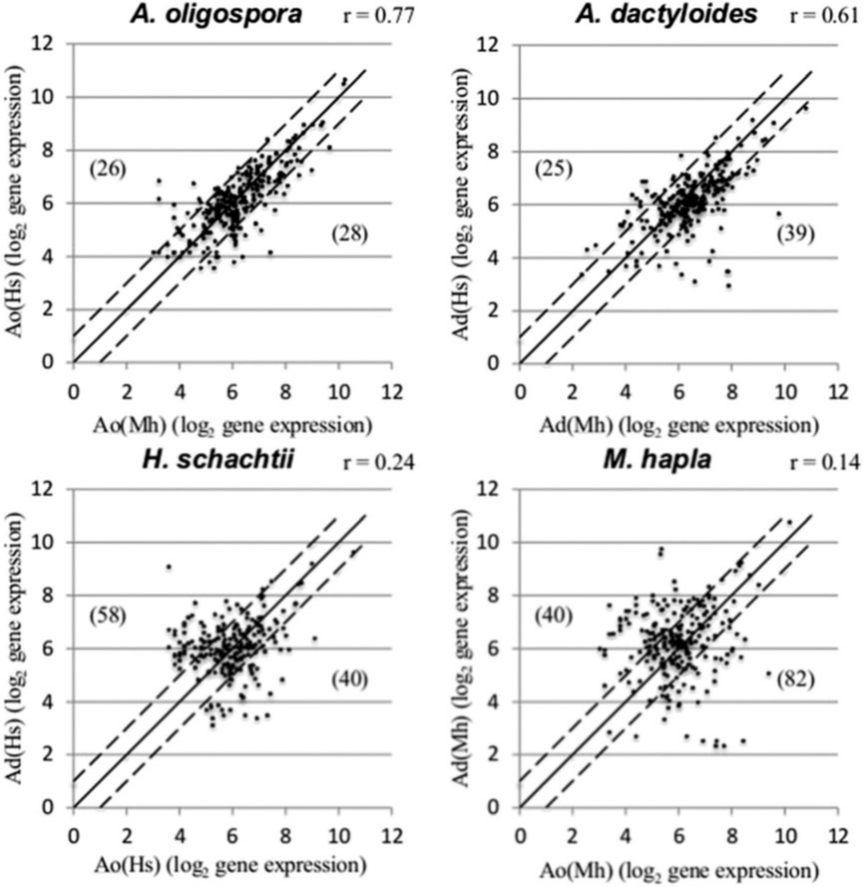
**Expression levels of highly expressed UniRef50 clusters.** Log_2_ scatter plot of gene expression pattern between *A. oligospora* and *A. dactyloides* infecting different nematodes (top) and *H. schachtii* and *M. hapla* infected by different fungi (bottom). The gene expression levels (normalized, log_2_-transformed read counts) of the 5% most highly expressed UniRef50 proteins represented by 326 unique IDs are shown. The Pearson correlation coefficients (r) of the comparisons are also shown. The diagonal line (y = x) shows UniRef50 clusters with nearly identical expression levels. The dotted lines correspond to a twofold expression difference. The numbers of clusters that differ in expression level more than twofold are shown in parentheses.

Analysis of the expression levels of the UniRef50 clusters confirmed these patterns. A PCA based on the 5% most highly expressed UniRef50 clusters showed that the first axis (49%) separated the two libraries of *A. dactyloides* from the two libraries of *A. oligospora* (Additional file 5). *M. cionopagum* was separated from the other libraries along the second axis (26%). Scatter plots showed that the numbers of UniRef50 clusters that were differentially expressed more than twofold between the fungal species were greater than between the host nematode species (Figure 4).

### Commonly expressed transcripts

In agreement with a previous study examining the infection-regulated transcriptome of *M. haptotylum* [20], the highly expressed transcripts of *A. dactyloides*, *A. oligospora* and *M. cionopagum* were enriched with those predicted to encode proteins with a secretion signal and those encoding orphans (Table 3). To characterize the core set of transcripts that was highly expressed by all fungi, the commonly expressed Pfam domains of *A. dactyloides*, *A. oligospora* and *M. cionopagum* were identified (Figure 3B). In total this set contained 206 Pfam domains that were annotated into putative functions (Table 4; Additional file 6). The core set of Pfam domains included several protein families that have previously been identified to be highly expressed by *A. oligospora* and *M. haptotylum* during infection of *C. briggsae*, such as subtilisin (peptidase_S8), aspartyl peptidases, CFEM (a fungal specific cysteine-rich domain that is found in some proteins with proposed roles in fungal pathogenesis) [28], and the carbohydrate-binding WSC domain [20, 22]. In addition, the core set contained a number of Pfam domains found in proteins involved in fungal stress response, cell signaling, organization of the cytoskeleton, vesicular transport and membrane transport, as well as several families of calcium-binding proteins and transcription factors. Domains of enzymes and proteins involved in the carbon, energy and amino-acid metabolism and protein synthesis were also highly expressed.

**Table 3:** **Proportion (%) of secreted proteins and orphans** ^**a**^

Category	Ao(Mh)	Ao(Hs)	Ad(Mh)	Ad(Hs)	Mc(Hs)
*Secreted proteins*					
All isotigs	8.4	9.4	7.8	8.6	9.1
Top 500 most expressed isotigs	12.2	12.6	12.6	10.8	13.8
(*P* ^b^)	(0.00031)	(0.00070)	(0.00002)	(0.01205)	(0.00008)
*Orphans* ^c^					
All isotigs	6.6	7.1	8.1	7.5	6.9
Top 500 most expressed isotigs	7.8	7.6	12.8	9.6	9.2
(*P* ^b^)	(0.03792)	(0.07609)	(0.00004)	(0.01176)	(0.00818)

^a^Proportion of secreted proteins and orphans among all identified isotigs (i.e. transcripts) and among the 500 most expressed isotigs in each sample. The samples are: *A. oligospora* infecting *M. hapla* (Ao(Mh)), *A. oligospora* infecting *H. schachtii* (Ao(Hs)), *A. dactyloides* infecting *M. hapla* (Ad(Mh)), *A. dactyloides* infecting *H. schachtii* (Ad(Hs)), and *M. cionopagum* infecting *H. schachtii* (Mc(Hs)).

^b^The probability (*P*) of observing a given number of isotigs within the functional category by chance using the hypergeometric distribution.

^c^Isotigs that lack known homologs and do not contain any Pfam domains.

**Table 4:** **Pfam domains expressed by all fungi during nematode infection** ^**a**^

Putative functions	Pfam domains
Peptidase	**Peptidase_S8** (PF00082); **Aspartyl protease** (PF00026); Peptidase_M3 (PF01432); Peptidase_S10 (PF00450);
Cell-surface proteins	**WSC** (PF01822)
Others	**CFEM** (PF05730); **Ricin-type lectin** (PF14200)
Stress response, chaperons	Thioredoxin (PF00085); Glutathione S-transferase (PF00043); Catalase (PF00199); AhpC/TSA family (PF00578); DnaJ (PF00226); HSP20 (PF00011); HSP70 (PF00012); HSP90 (PF00183); Peptidylprolyl isomerase (PF00254, PF00160)
Cell signaling	RHO protein GDP dissociation inhibitor (PF02115); ADP Ribosylation Factors (ARFs) (PF00025); 14-3-3- proteins (PF00244); Pkinase (PF00069); Ras (PF00071)
Calcium-binding protein	Calreticulin (PF00262); EF-hand motif (PF13499)
Cytoskeleton	Actin (PF00022); Cofilin (PF00241); Profilin (PF00235); Tropomyosin (PF12718); Tubulin (PF00091)
Autophagy	Atg8 (PF02991)
Peroxisome	Membrane protein Mpv17_PMP22 (PF04117)
Trancription	Multiprotein bridging factor 1 (PF08523); Homeobox (PF00046); TATA binding protein (PF00352); bZIP Transcription factor (PF00170, PF07716); Histone (PF00125, PF00538); Nucleosome assembly protein (PF00956); Helicases (PF00270, PF00271)
Membrane transport	ABC transporter (PF00005) Amino acids permease (PF00324); Ammonium transporter (PF00909); Major Facilitator Superfamily (PF07690); Sugar and others (PF00083); Porin (PF01459)
Vesicular transport	Syntaxin (PF05739); Synaptobrevin (PF00957); Rab GDP dissociation inhibitors (PF00996)
Metabolism, glycolysis/gluconeogenesis	Enolase (PF03952); Fructose-bisphosphate aldolase (PF01116); Triose-phosphate isomerase (PF00121); Glyceraldehyde 3-phosphate dehydrogenase (PF00044); Phosphoglycerate kinase (PF00162); Pyruvate kinase (PF00224); Pyruvate carboxylase (PF00682)
Metabolism, pentose phosphate pathway	Phosphogluconate dehydrogenase (PF03446, PF00479); Transaldolase (PF00923); Transketolase (PF00456)
Metabolism, TCA	lactate/malate dehydrogenase (PF02866, PF00056); Succinyl coenzyme A synthetase (PF00549)
Metabolism, energy	Cyt-b5 (PF00173); ATP synthase (PF00887, PF00137); mitochondrial carrier (PF00153); ATPases (PF00006, PF02874)
Metabolism, amino acids	Glutamine amidotransferases (PF00310); NAD-specific glutamate dehydrogenase (PF10712); Glutamine synthetase (PF03951)
Metabolism, miscellaneous	Glycoside hydrolase family 1 (PF00232); Biotin-requiring enzyme (PF00364) aldo-keto reductase family (PF00248); Aldehyde dehydrogenase family (PF00171); Short-chain dehydrogenases/reductases family (PF00106); Pyrophosphatase (PF00719); Myo-inositol-1-phosphate synthase(PF01658, PF07994); FA desaturase (PF00487); Nucleoside diphosphate kinase (PF00334); UDP-glucose pyrophosphorylase (PF01704); Transketolase, pyrimidine binding domain (PF02779); ATPase (PF00004); CoA binding domain (PF02629) ATP-grasp_2 (PF08442); Calcineurin-like phosphoesterase (PF00149); Epimerase (PF01370)
Protein degradation	Proteasome (PF00227, PF10584); Ubiquitination (PF00240, PF00240, PF00179)
Protein synthesis	Elongation factors (PF10587, PF01873, PF00009); tRNA synthetases (PF00587); translation, initiation factors W2 (PF02020), SUI1 (PF01253), MIF4G (PF02854); Ribosomal proteins (PF00428, PF00428, PF00687, PF00466, PF00238, PF00827, PF00252, PF14204, PF01775,PF00828, PF00861, PF01280, PF03947, PF01157, PF01776, PF01246, PF01777, PF00831, PF00297, PF01198, PF01655, PF01780, PF01907, PF01020, PF00935, PF00281, PF00347, PF01159, PF01248, PF00411, PF00164, PF00416, PF00253, PF00833, PF00203, PF01090, PF00318, PF01282, PF03297,PF01283, PF01667, PF01015, PF00163, PF00333, PF00177, PF00410, PF01201, PF00380, PF08071)

^a^Shown are Pfam domains that were found among the top 500 most expressed isotigs in at least one library of each fungus and that are shared between all three fungi. In total 206 Pfam domains were found in this cohort (*c.f.* Figure 3B; Additional file 6). Pfam domains encoding protein motifs with unspecific or unknown functions are not shown. Bold indicates domains that were found among 25 expanded Pfam domains identified in the genome of *M. haptotylum* [20].

Furthermore, transcripts containing the Atg8 domain were highly expressed in all fungi. Atg8 is an essential protein in the autophagic pathway [29] and disruption of a homolog of this gene in *A. oligospora* leads to reduced trap formation [30]. In addition, all three fungi expressed transcripts with the Pfam domain RicinB_lectin_2 (PF14200). Ricin-B lectins are ribosome-inactivating proteins (RIPs) consisting of a catalytic A-chain and a sugar-binding B-chain [31, 32]. All fungi have a RicinB_lectin_2 transcript that match to G1X3G7 in *A. oligospora*. G1X3G7 is a protein with a length of 134 amino-acid residues (aa), without a secretion signal and with low sequence similarity to other RicinB lectins in the UniProt database. A RicinB_lectin_2 domain-containing protein (MOA) with nematotoxic activity against *Caenorhabditis elegans* has been identified in the basidiomycete *Marasmius oreades* [33]. The nematotoxicity was dependant on the cysteine protease activity of MOA and the binding of its lectin domain to glycosphingolipids in the worm intestine. MOA consists of 293 aa and lacks a classical secretion signal [33]. *Sclerotinia sclerotiorum* agglutinin (SSA) is a RicinB_lectin_2 domain-containing protein with a length more similar to the G1X3G7 protein in *A. oligospora* [34]. SSA has a length of 153 aa, lacks secretion signal and shows insecticidal properties when fed to the pea aphid *Acyrthosiphon pisum* [34].

Previous studies have shown that subtilisins (peptidase_S8) are important virulence factors in nematode-trapping fungi. In *A. oligospora* they have a key role in the early stages of infection, including immobilization of the captured nematode [19, 35, 36]. *A. oligospora* has 52 genes containing the peptidase_S8 domain [20]. However, only one transcript containing the peptidase_S8 domain was identified among the highly expressed transcripts in each library of Ao(Mh) and Ao(Hs). BLASTX searches showed that both transcripts displayed the highest sequence homology to the *A. oligospora* protein G1XLL2. The other cDNA libraries contained also only one transcript with the peptidase_S8 domain among the top 500 expressed genes. The three transcripts in Ad(Mh), Ad(Hs) and Mc(Hs) all displayed the highest sequence homology to H072_8474 in *M. haptotylum*. Interestingly, G1XLL2 and H072_8474 are orthologs (T. Meerupati, B. Canbäck, D. Ahrén, A. Tunlid, manuscript in preparation). Furthermore, G1XLL2 was the most expressed peptidase_S8 gene in *A. oligospora* during early infection (6 and 10 hours) of *C. briggsae*, and H072_8474 was the second most expressed peptidase_S8 gene in *M. haptotylum* during early infection (4 hours) of *C. briggsae* [20]. H072_8474 was also identified in the proteome of both the knob and the mycelia in *M. haptotylum* [22]. This shows that despite the large number of peptidase_S8 genes only a few are highly expressed during infection.

Stress proteins were highly expressed in all fungi independent of trapping structure. They included heat-shock proteins and chaperones such as DnaJ, HSP70 and HSP90; gluthatione S-transferases; and antioxidant enzymes such as thioredoxin and catalase. Antioxidants are enzymes involved in the protection of the cell from oxidative damages induced by reactive oxygen species (ROS) [37]. ROS are continuously produced in the cell as byproducts from various metabolic pathways and have an important role(s) in signaling [38]. In the plant-pathogenic fungus *Magnaporthe grisea*, ROS-generating NADPH oxidases (Nox1 and Nox2) are essential for pathogenicity [39]. The authors [39] suggest that the generated ROS accumulate in the appressorium to facilitate oxidative cross-linking of cell-wall proteins. This leads to a strengthening of the cell wall of the appressorium that will eventually resist high turgor pressure [39]. Transcripts with sequence similarity to the Nox proteins in *M. grisea* were regulated in all fungi of our study during infection. However, none of them were found among our top 500 most expressed transcripts.

### Interspecific variation in gene expression

**Figure 5:**
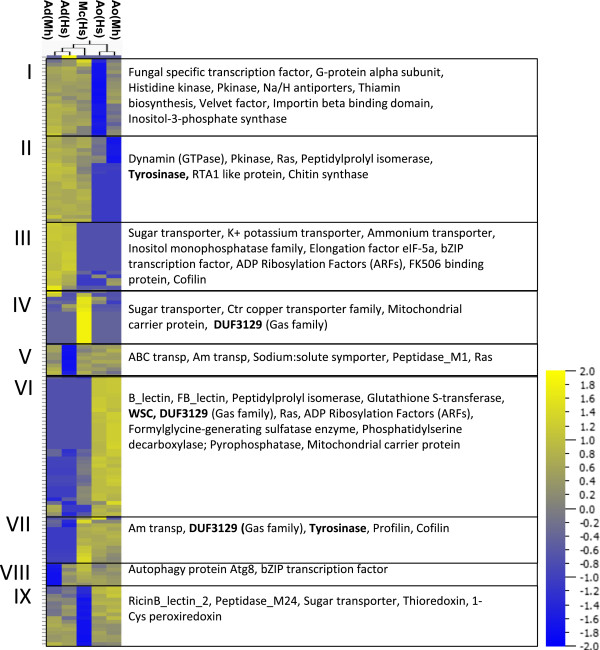
**Heat map of gene expression levels of UniRef50 clusters.** Gene expression levels of the 5% most highly expressed UniRef50 clusters passing a variance filtering of 0.3 (total 160) are shown. I to IX indicate nine clusters that were identified using hierarchical clustering of normalized, log_2_-transformed read counts (+1). The right panels shows annotations of the UniRef50 cluster sequences based on the presence of Pfam domains (Additional file 7). Bold indicates domains that were found among 25 expanded Pfam domains identified in the genome of *M. haptotylum*[20]. Ao(Mh) denotes *A. oligospora* and *M. hapla*; Ao(Hs), *A. oligospora* and *H. schachtii*; Ad(Mh), *A. dactyloides* and *M. hapla*; Ad(Hs), *A. dactyloides* and *H. schachtii*; and Mc(Hs), *M. cionopagum* and *H. schachtii*.

To identify the variable sets of transcripts, that is, the transcripts that were differentially regulated depending on the fungal species, the expression levels of the putative orthologs identified using the UniRef50 clusters were compared among the libraries (Figure 5; Additional file 7). Among these were transcripts encoding peptidases (peptidase_M1 and peptidase_M24), lectins (FB_lectin, B_lectin, RicinB_lectin_2), tyrosinase, transcription factors, cell-signaling components, Atg8, various stress response proteins, proteins containing the WSC domain and the DUF3129 domain. DUF3129 is a domain of unknown function that is found in the gas1 protein of *M. grisea*, which participates in appressorial penetration and lesion formation [40]. Interestingly, DUF3129 was highly expressed in *M. cionopagum* but not expressed at all in *A. dactyloides*. DUF3129 was identified in 12 transcripts among the top 500 most expressed transcripts in the Mc(Hs) library. During *A. oligospora* infections, this domain was identified in one transcript in Ao(Mh) and in four transcripts in Ao(Hs) among the top 500 most expressed transcripts. DUF3129 is an expanded gene family in nematode-trapping fungi and both *M. haptotylum* and *A. oligospora* have 33 genes encoding this domain [20]. Seventeen of these genes were previously found among the 10% most expressed genes during nematode infection by *M. haptotylum*, whereas only two were among the 10% most expressed genes during nematode infection by *A. oligospora* [20]. The DUF3129 domain is thus highly expressed during infection among the species that form adhesive branches and adhesive knobs. Further studies are needed to investigate the function of the DUF3129 domain in the nematode-trapping fungi during infection.

Transcripts encoding the fungal fruit-body lectin (FB_lectin) and the D-mannose binding lectin (B_lectin) were only highly expressed in *A. oligospora* and not in the other two fungi (Figure 5). In the UniRef50 cluster containing the FB_lectin domain we identified the previously studied AOL lectin (Q00233) [41]. The transcript matching to this cluster was the third most expressed of all transcripts during Ao(Mh) infection and the 25th most expressed of all transcripts during Ao(Hs) infection. Earlier studies have shown that AOL functions as a storage protein during both saprophytic and parasitic growth [42]. However, deletion of this gene did not affect the fungus’ ability to infect nematodes [43].

Previous studies have shown that proteins containing the carbohydrate-binding domain WSC comprise a large and rapidly evolving gene family in *M. haptotylum* [22]. Phylogenetic analysis of the 33 WSC-containing proteins in *M. haptotylum* revealed a clade of 15 WSC paralogs [22]. This clade contains only one (G1X6Q5) of the 16 WSC proteins identified in *A. oligospora* [22]. Thirteen of the 15 WSC paralogs of *M. haptotylum* were at least twofold upregulated during the infection of the nematode *C. briggsae* [20] *.* In this study, transcripts encoding WSC proteins were highly expressed by all fungi during infection of plant-parasitic nematodes (Table 4). The largest number of transcripts encoding WSC domain proteins was expressed by *M. cionopagum*. In total, 19 transcripts of WSC domain proteins were identified in the Mc(Hs) library, of which seven were found among the top 500 transcripts. Four of these transcripts displayed closest sequence homology to proteins found in the expanded clade of WSC proteins of *M. haptotylum* [22]. The libraries of *A. oligospora* and *A. dactyloides* contained a lower number of transcripts of WSC domain proteins. Among the top 500 transcripts, the Ao(Hs), Ao(Mh) and Ad(Mh) libraries each had two WSC domain proteins, whereas none were found in the Ad(Hs) library. Transcripts displaying highest sequence homology to the previously mentioned *A. oligospora* protein G1X6Q5 were identified in both the Ao(Hs) and the Ao(Mh) libraries. The deduced proteins of two highly expressed WSC transcripts in *A. dactyloides* did not show any sequence similarity to the proteins found in the expanded clade of paralogs in *M. haptotylum* [22]. Taken together, the comparative transcriptome analysis shows that the WSC domain proteins comprise a large and divergent gene family that is highly expressed during pathogenesis in nematode-trapping fungi. The specific sets of genes that are expressed depend on the fungal species and the nematodes being infected, which suggests that the function of the WSC proteins is to contribute to the specialization of the trapping mechanisms.

### Virulence associated transcripts

A BLAST search of the top 500 transcripts in each library was conducted in the pathogen–host interaction protein database (PHI-base) [44]. PHI-base contains experimentally verified pathogenicity, virulence and effector genes from fungi, oomycetes and bacterial pathogens. In total, 97 unique PHI-base genes were identified. Genes with sequence similarity to seven of them were found in at least ten gene models in either *M. haptotylum* or *A. oligospora* [20]. They included *RBT4* from *Candida albicans*, which is necessary for virulence [45]. The function of *RBT4* is unknown but it contains a CAP (Cysteine-rich secretory proteins, Antigen 5 and Pathogenesis-related 1 protein) domain [45]. Among the 97 identified PHI-base genes, 15 were highly expressed by all fungal species (Additional files 8 and 9) and 82 were highly expressed by one or two of the fungal species (Additional file 10). The PHI-base genes expressed by all fungi included stress response genes and several cell signaling genes containing the Ras domain. The PHI-base genes that differ in expression between the fungi included aspartic proteases and the gas 1 and gas 2 proteins of *M. grisea* [40] that contains the DUF3129 domain.

### Host-specific gene expression

**Figure 6:**
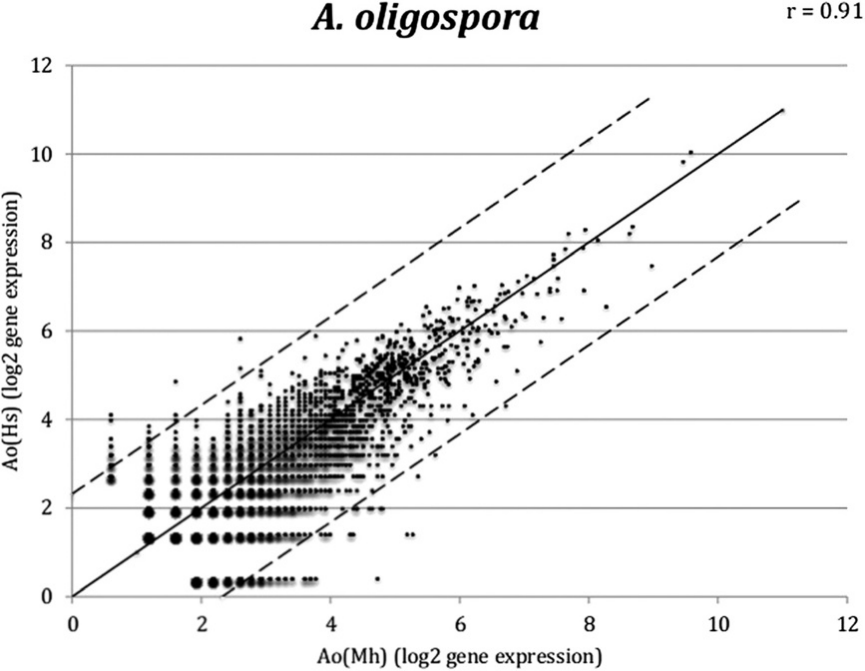
**Host-specific gene expression in** ***Arthrobotrys oligospora*** **.** Scatter plot of normalized mapped reads of *A. oligospora* and *H. schachtii* (Ao(Hs)) versus *A. oligospora* and *M. hapla* (Ao(Mh)). Genes that were regulated (≥1 read) in both libraries and that had ≥5 reads in any of the libraries were included in the analysis, 4 138 genes in total. The Pearson correlation coefficient (r) of the comparison is also shown. The diagonal line (y = x) shows transcripts with near identical expression levels. The dotted lines correspond to a five-fold expression difference.

A scatter plot of the gene expression in *A. oligospora* during the infection of *M. hapla versus H. schachtii* showed that a majority of the genes had similar expression levels (Figure 6). However, 105 genes were expressed at levels at least 5-fold higher during infection of *M. hapla* than during infection of *H. schachtii* (Additional file 11), and 65 genes were expressed at levels at least 5-fold higher in *H. schachtii* than in *M. hapla* (Additional file 12). Genes predicted to encode secreted proteins were enriched among the differentially expressed genes in both nematodes. The proportion of secreted proteins among the upregulated genes in *M. hapla* (Ao(Mh) or Ad(Mh)) was 12.4% (13 out of 105) and in *H. schachtii* (Ao(Hs) or Ad(Hs)) was 13.8% (9 out of 65). In comparison, the proportion of secreted proteins among all genes that were used for the host-specific gene expression analysis was 7.3% (304 out of 4,138).

The differentially expressed genes encoding proteins with a predicted secretion signal were further characterized (Table 5). These included peptidases and several gene families that were expanded in the genomes of nematode-trapping fungi, such as DUF3129, WSC and tyrosinase [20]. One chitinase was identified that contains a LysM domain. Chitinases with LysM domains (CBM50) have been shown to have sequence similarity to the yeast killer toxin of *Kluyveromyces lactis* [46]. Five of the 22 secreted differentially expressed genes were assigned as SSPs with unknown function (Table 5).

**Table 5:** **Differentially expressed genes encoding secreted proteins in** ***A. oligospora*** **during infection of** ***M. hapla*** **or** ***H. schachtii*** ^**a**^

UniProt ID	Description ^b^	Pfam	Length ^c^	Fold change
*Upregulated in M. hapla*				
G1X4P0	Histidine acid phosphatase	His_Phos_2	476	9.1
G1XTC9	Patched sphingolipid transporter	Patched	1 292	9.1
G1XF88	Uncharacterized protein	-	251^e^	7.7
G1XGH3	IgE-binding protein	-	195	7.4
G1XHE8^d^	-	-	196^e^	6.9
G1X1U4	Glucosidase 2 subunit beta	-	553	6.3
G1X8R3	GPI anchored cell-wall protein	But2	328	6.2
G1XCM7	Beta-galactosidase	Glyco_hydro_35, BetaGal_ dom2, BetaGal_dom3, BetaGal_dom4_5	982	5.7
G1XET4	Peptidase S41	Peptidase_S41	702	5.7
G1XGI5	Uncharacterized protein	-	107^e^	5.3
G1XPV3	Uncharacterized protein	-	173^e^	5.3
G1XJP0	Uncharacterized protein	-	606	5.1
G1XLE5	Aminopeptidase Y	PA, Peptidase_M28	503	5.1
*Upregulated in H. schachtii*				
G1X7Q6^d^	-	-	300	15.8
G1XQA9	Uncharacterized protein	-	707	15.8
G1XU57	ABC transporter	ABC_tran, ABC2_membrane	1 047	15.8
G1X110^d^	-	-	151^e^	11.4
G1XR64	Uncharacterized protein	DUF3129	656	10.5
G1XF27	WSC-domain-containing protein	WSC	625	9.6
G1XEV7	Chitinase	Glyco_hydro_18, LysM	1 232	8.8
G1XM84	Tyrosinase	Tyrosinase	387	8.8
G1XC55	Uncharacterized protein	DUF3129	454	6.4

^a^Genes encoding proteins with a predicted secretion signal that were upregulated at least 5-fold in the sample *A. oligospora* infecting *M. hapla* (Ao(Mh)) compared to the sample *A. oligospora* infecting *H. schachtii* (Ao(Hs)) and in Ao(Hs) compared to Ao(Mh). Genes that were expressed (≥1 read) in both libraries and that had ≥5 read in any of the libraries were included in the analysis, in total 4 138 genes.

^b^Non-annotated genes of *A. oligospora* were further characterized by searches through the UniProt database [47] using the BLASTP algorithm [25] with an E-value threshold of 1e-10. Fungal sequences were chosen among the top hits.

^c^The length of the protein sequence in aa.

^d^Orphan, protein that lacks known homologs and does not contain any Pfam domains.

^e^Small secreted protein (SSP) with unknown function. SSPs were defined as secreted proteins with a length of less than 300 aa.

## Conclusions

This is the first study examining the variation in gene expression patterns among nematode-trapping fungi during infection of different host species. Comparative transcriptome analysis showed that the divergence in gene expression between the fungal species was significantly larger than that related to the nematode host. A core set of transcripts that were highly expressed by all three fungi was identified. This core set included subtilisins, aspartic proteases and proteins containing the CFEM domain. These genes were also highly expressed by *A. oligospora* and *M. haptotylum* during infection of *C. briggsae* [20]. Also within this core set, a putative nematotoxic protein was identified, the Ricin-B lectin. A more variable set of transcripts being regulated depending on the fungal species was also identified. A small set of genes was identified showing differential expression depending on the host. This set was enriched in genes encoding secreted proteins and also included several gene families that were expanded in the genomes of nematode-trapping fungi [20]. Information on the genetic basis of the interspecific variation in the trapping mechanisms and host preferences fungi will be useful for researchers who are screening for more potent biological control agents of nematode-trapping fungi.

## Methods

### Culture of organisms and infection experiments

Cultures of *A. oligospora* (ATCC 24927), *M. cionopagum* (CBS 220.54) and *A. dactyloides* (CBS 109.37) were maintained on corn meal agar 1:10. Infested soil/roots of *M. hapla* (Strain E 226) were obtained from Prof. Dr. Gerrit Karssen (Plant Protection Service, HC Wageningen, the Netherlands) to start the culture of this nematode. Small pieces of roots infested with *M. hapla* were inoculated in rhizosphere of 3-week-old tomato plants raised in the green house of Department of Biology, Lund University, for induction of root knots. After 8 weeks, the infected tomato roots with well developed knots and egg masses were gently washed under running tap water. Egg masses of *M. hapla* were picked by fine forceps from knots of infected roots under a stereoscopic binocular microscope and surface disinfected for 1 minute in a 0.5% NaOCl solution and rinsed three times in sterile distilled water. Egg masses were then collected in Petri dishes (30 mm) in sterile distilled water and incubated at 22 ± 1°C for 48 hours for hatching of second-stage juveniles (J_2_). After incubation, freshly hatched J_2_s were separated from the egg masses and collated in Eppendorf tubes, surface sterilized with 0.5% NaOCl for 2 minutes, and rinsed five times with sterilized distilled water, and used for infection experiments. J_2_s of *H. schachtii* were obtained from HZPC in the Netherlands (http://www.hzpc.com) and used for infection experiments after sterilization and washing as described for *M. hapla.*

Infection experiments were performed using a dialysis membrane assay [48]. Briefly, conidia of *A. oligospora*, *M. cionopagum* and *A. dactyloides* were inoculated onto several pieces of sterilized dialysis membrane (spectra/por 4, Spectrumlabs). The membranes were placed over plates containing modified low-nutrient mineral salt (LNM) medium (KCl 1.0 g/l, MgSO_4_ 0.2 g/l, ZnSO_4_.7H_2_O 0.88 mg/l, FeCl_3_.6H_2_O 3.0 mg/l, thiamine-HCl 0.2 mg/l, biotin 0.005 mg/l, L-phenylalanine-L-valine 0.1 g/l, agar 10 g/l, pH 6.5) [48, 49]. Infection structures (traps) were induced by adding 40–50 specimens of the nematode *Panagrellus redivivus* L. (Goodey) to the hyphae growing on each dialysis membrane. *P. redivivus* was grown axenically in a soya peptone-liver extract [50]. After several days, when substantial amount of traps have been developed and all added nematodes have been killed and digested, the infection experiments were started by adding 75–100 surface sterilized second-stage juveniles of *M. hapla* or *H. schachtii*. The following five combinations of fungi and nematodes were examined: *A. oligospora* and *M. hapla* (designated Ao(Mh)), *A. oligospora* and *H. schachtii* (Ao(Hs)), *A. dactyloides* and *M. hapla* (Ad(Mh)), *A. dactyloides* and *H. schachtii* (Ad(Hs)), and *M. cionopagum* and *H. schachtii* (Mc(Hs)). The infection was followed under a light microscope, and the number of trapped, paralyzed (i.e. with arrested movements), and colonized (hyphae growing inside the capture nematode) were counted after various time periods. For each fungal and nematode interaction, 10 replications were used. Dialysis membranes having fungal and nematode interaction of each stage were quickly transferred into liquid nitrogen and ground. Materials were collected from all infection stages (trapped, paralyzed and infected (colonized) nematodes). The ground material was stored at -80°C until use.

### RNA extraction, cDNA library construction and sequencing

Total RNA was extracted from each infection stage using the RNeasy Plant Mini kit and the RLC buffer (Qiagen) and subsequently quantified using a NanoDrop 2000C spectrophotometer (Thermo Scientific). RNA integrity was inspected using a RNA 6000 Pico kit on a 2100 BioAnalyzer (Agilent). Approximately equal amounts of RNA from the three infection stages of each fungus-nematode combination were pooled. The RNA pools were concentrated by precipitation using ammonium acetate/glycogen/ethanol as described in the MicroPoly(A)Purist Kit manual (Ambion).

From total RNA, mRNA was isolated using the PolyATtract kit (Promega) according to manufacturer’s instructions. Double-stranded cDNA was synthesized using the cDNA Synthesis System (Roche Diagnostics) according to the GS FLX Titanium cDNA Rapid Library preparation protocol (454/Roche) and using adaptors with Multiplex Identifiers (MIDs) that allow for pooling of the libraries prior sequencing. Library concentration was assessed by qPCR on a Mx3005P instrument (Stratagene) and using the KAPA Library Quantification Kit - 454 Titanium (Lib-L)/Universal (Kapa Biosystems). Based on the qPCR results all libraries were pooled to contain an equal molar amount of each library. Titration and library production (aiming at 7-16% enrichment) was performed using emulsion PCR and the Lib-L kit (454/Roche). DNA-containing beads were enriched and counted using a CASY Cell Counter DT (Roche Innovatis AG), processed using aXLR70 sequencing kit (454 Life Sciences/Roche Diagnostics), and loaded onto a picotiter plate for pyrosequencing on a 454 Life Sciences Genome Sequencer FLX machine (454 Life Sciences/Roche Diagnostics). Sequencing was conducted at the Lund University Sequencing Facility (Faculty of Science).

### Bioinformatic analyses

The reads obtained from the 454 sequencing were filtered, assembled and analysed according to the flowchart shown in Figure 2. Reads matching rRNA were removed using the BLASTN algorithm [25] with an E-value threshold of 1e-5 against a custom made database of rRNA sequences obtained from the 5S rRNA database [51] and the SILVA rRNA database [52]. The remaining reads for each of the five libraries were assembled separately using the GS *de novo* assembler 2.6 (454 Life Sciences/Roche Diagnostics) with the *-cdna* option. The reads were assembled into 17 785 isotigs and 10 contigs with a length > 500 bp. In the following text, both categories were referred as isotigs, *i.e.* transcripts. Low abundance isotigs with less than five reads and isotigs with a length shorter than 100 bp were removed. Isotigs with top hits to non-fungal species in the UniProt database [47] (the BLASTX search) [25] were also removed. The filtered dataset contained 17 446 isotigs.

The filtered isotigs from the two *A. oligospora* samples were mapped to the *A. oligospora* genome using Gmap [53] to assess the quality of the *de novo* isotig assemblies. In total, 3 944 out of 3 952 isotigs (including 2 634 istotigs from the Ao(Mh) library and 1 318 isotigs from the Ao(Hs) library, Table 2)) matched the genome indicating efficient filtering of non-fungal transcripts. Only 75 of the 3 944 isotigs aligned to more than one position in the genome giving a total of 4 019 genome regions aligning to the isotigs. The low number of isotigs with multiple matches indicates a low frequency of chimeric transcripts. To further investigate the quality of the transcriptome assembly, we compared the genome sequences of isotig alignments with the 11 479 predicted genes from the *A. oligospora* genome using the Eval software [54]. All of the 4 019 genome regions matched to the predicted *A. oligospora* genes. In total, 2 652 *A. oligospora* genes were matched giving on average 1.5 isotigs per predicted gene which suggest that some of the isotigs may represent alternative splicing forms. In total, 94.6 percent of the aligned genome regions (3 803 out of 4 019) contained both start and stop codon (i.e. considered complete by the Eval software). The mean length of the isotigs was 1 109 basepairs compared to 1 498 basepairs for the *A. oligospora* gene models. The difference in length may at least partly be due to alternative splicing forms where exon skipping will give shorter transcripts than the predicted gene models. The high proportion of successful matches to the *A. oligospora* genome as well as to its genes, the large number of complete isotigs and the long mean isotig length clearly indicate that most of the filtered isotigs have been correctly assembled into near full length transcripts.

The filtered isotigs were used to generate two different data sets (Figure 2). The first data set (“Highly expressed transcripts”) was normalized using two different approaches; reads per kilobase pair (kb), (the number of aligned reads per transcript was divided by the transcript length), and the reads per kilobase per million reads (RPKM) method [55] (Additional file 1). The isotigs were annotated based on homology using the BLASTX algorithm [25] (threshold values of 1e-10) to the UniProt sequence database [47] and proteins of *M. haptotylum* [20]. The isotigs were also annotated using the pfam_scan.pl tool (ftp://ftp.sanger.ac.uk/pub/databases/Pfam/Tools/) to search the Pfam-A family protein database [56] with default thresholds. Secretion signals were predicted using the SignalP 4.0 algorithm [57]. Isotigs were considered to encode putative secreted proteins if fulfilling at least one of the following three criteria: 1) Isotigs having a secretion signal in the same frame as the Pfam domain; 2) Isotigs having a secretion signal in the longest predicted open-reading frame (ORF) in the same frame as the BLASTX match (threshold value of 1e-10) to a protein in the UniProt database or protein from *M. haptotylum;* 3) Isotigs having a BLASTX match to a protein in the UniProt database or protein from *M. haptotylum* that contains a secretion signal. Orphans were identified as isotigs lacking both Pfam domains and BLASTX matches in the UniProt database and the *M. haptotylum* genome (threshold value of 1e-5) against a species other than itself. Orphans with a secretion signal in the longest ORF were considered to be putative secreted proteins. PCA and hierarchical clustering were performed using the Omics Explorer ver. 2.2 (Qlucore). Virulence-related genes were identified by BLASTX [25] similarity searches against the PHI-base database version 3.2 [44] using a cutoff of < 1e-10.

The second data set (“Differentially expressed UniRef50 clusters”) was obtained by matching the isotig sequences to UniRef50 clusters [26]. The UniRef50 clusters contain a representative of UniProt sequences that show 50% sequence similarity and 80% overlap with the longest sequence in the cluster. The isotigs with the highest BLASTX score to a UniRef50 sequence cluster from each library were considered as putative orthologs giving maximum one isotig from each species for any given UniRef50 cluster to take alternative splicing into account. Sometimes no isotig from one species has a significant match to a particular UniRef50 cluster (cutoff 1e-10). The transcript abundance of these putative orthologs, called “UniRef50 clusters” was normalized to correct for different library sizes using the R/Bioconductor software package DESeq 1.10.1 [27] (Additional file 2).

To identify the third data set, “Host-specific gene expression” (Figure 2), GSMapper 2.8 (454 Life Sciences/Roche Diagnostics) was used with the *-cdna* and -*cref* parameters to map the reads from the two *A. oligospora* libraries (Ao(Mh) and Ao(Hs)) against the coding sequences of the 11 479 genes predicted in the *A. oligospora* genome [19]. The read counts were normalized using DESeq [27]. A homology search of the mapped *A. oligospora* proteins was carried out using the BLASTP algorithm [25] to the UniProt database and proteins of *M. haptotylum* (threshold values of 1e-5). Secretory *A. oligospora* proteins were predicted using SignalP 4.0 [57].

### Sequence accession numbers

Sequences can be accessed from the database http://mbio-serv2.mbioekol.lu.se/NematodeTrappingFungi/. The short read pyrosequences from Ad(Mh), Ad(Hs), Ao(Mh), Ao(Hs) and Mc(Hs) are available at NCBI SRA database with the Bioproject IDs PRJNA230433, PRJNA230458, PRJNA230459, PRJNA230446 and PRJNA230448, respectively.

## Electronic supplementary material

Additional file 1: **Evaluation of procedures used for normalizing sequence reads.** (PDF 141 KB)

Additional file 2: **Evaluations of procedures used for normalizing gene expression levels of UniRef50 clusters.** (PDF 211 KB)

Additional file 3: **Summary of the annotation of the 500 most expressed transcripts.** (PDF 94 KB)

Additional file 4: **Annotation of the 500 most expressed transcripts.** (XLSX 256 KB)

Additional file 5: **PCA analysis of highly expressed UniRef50 clusters.** (PDF 94 KB)

Additional file 6: **Number of pfam domains expressed by all fungi during nematode infection.** (XLSX 21 KB)

Additional file 7: **Isotigs and Pfam annotations of UniRef50 clusters shown in the heatmap of Figure** 5. (XLSX 24 KB)

Additional file 8: **Isotigs displaying sequence similarity to proteins in the PHI-database.** (XLSX 31 KB)

Additional file 9: **Virulence associated proteins highly expressed by all fungi.** (PDF 165 KB)

Additional file 10: **Variations in expression of virulence associated proteins.** (PDF 68 KB)

Additional file 11: **Upregulated genes in** ***A. oligospora*** **during infection of** ***M. hapla*** **as compared with** ***H. schachtii.*** (PDF 104 KB)

Additional file 12: **Upregulated genes in** ***A. oligospora*** **during infection of** ***H. schachtii*** **as compared with** ***M. hapla.*** (PDF 85 KB)
