# Supplementary material for: Interspecific and host-related gene expression patterns in nematode-trapping fungi
Source: BMC Genomics. 2014 Nov 11;15(1):968. doi: 10.1186/1471-2164-15-968 (PMC4237727; doi:10.1186/1471-2164-15-968)
Supplement: Supplementary file 1 — Additional file 1: Evaluation of procedures used for normalizing sequence reads. (PDF 141 KB) [file 12864_2014_6662_MOESM1_ESM.pdf]

Additional file 1. Evaluation of procedures used for normalizing sequence reads

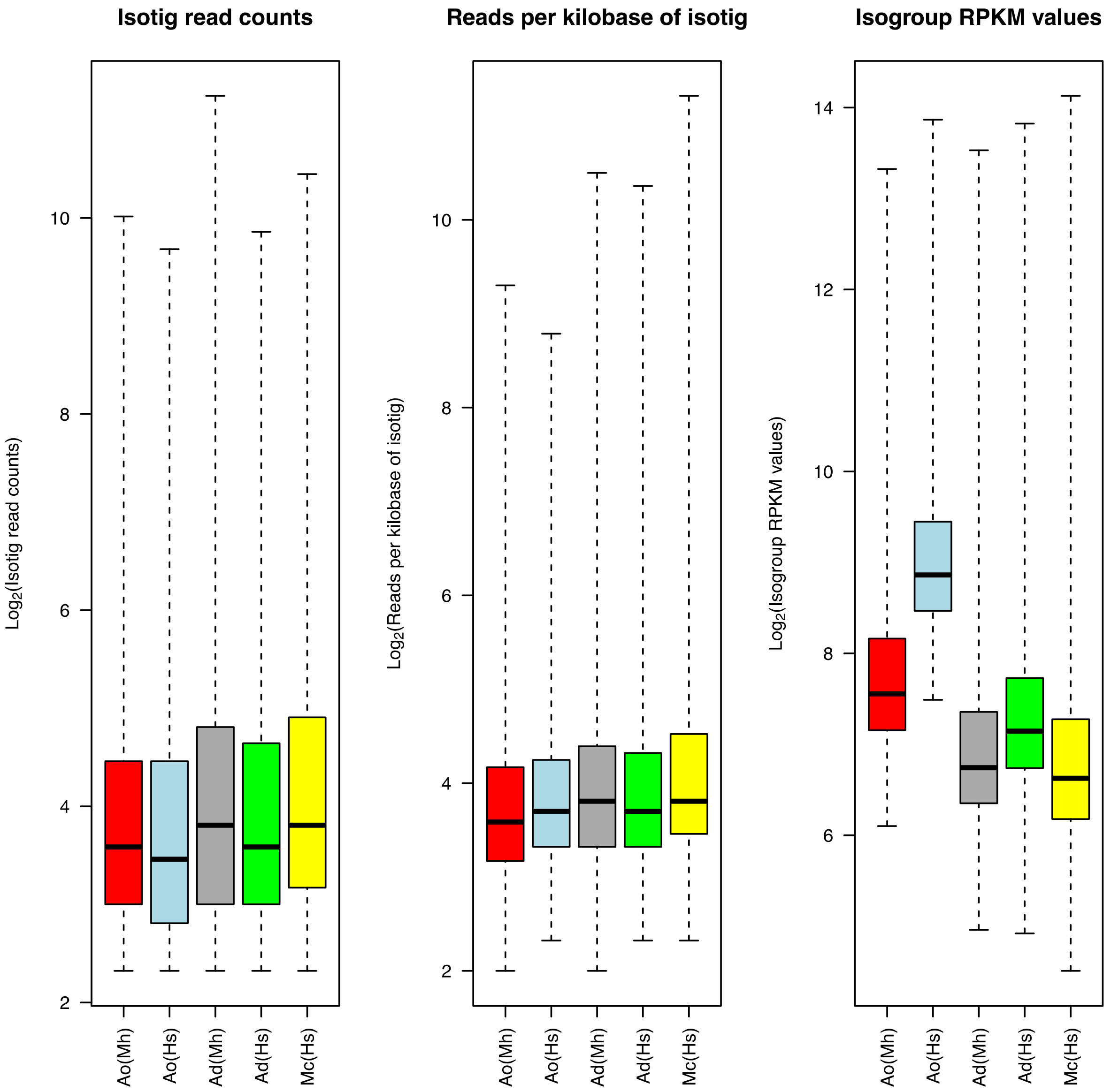

The left-side panel shows isotig raw read counts, the middle panel shows isotig-length normalized read counts and right-side panel shows isogroup read counts normalized with the Reads Per Kilobase per Million reads (RPKM) method [1]. The line in the box represents the median of the read distribution whereas the bottom and the top of the box represent the 25th percentile and the 75th percentile, respectively. The minimum and maximum values are indicated by the ends of the vertical lines. Outliers are not shown in the box plots.

Ao(Mh) denotes *A. oligospora* and *M. hapla*; Ao(Hs), *A. oligospora* and *H. schachtii*; Ad(Mh), *A. dactyloides* and *M. hapla*; Ad(Hs), *A. dactyloides* and *H. schachtii*; and Mc(Hs), *M. cionopagum* and *H. schachtii*.

The RPKM value of each isogroup was calculated as $RPKM = (C \times 10^6) / (L \times N)$ where *C* is the read count per isogroup, *L* is the isogroup length in kb and *N* is the total number of reads that were aligned to the isogroups. A Perl script was used to calculate the isogroup lengths

and read counts using the 454Isotigs.fna, 454Isotigs.ace, and 454AllContigs.fna files from GSAssembler (454 Life Sciences/Roche Diagnostics) as input. The isogroup length was calculated as the sum of all contig lengths within the isogroup. The read counts per isogroup were calculated as the sum of uniquely aligned reads per isogroup.

References

1. Mortazavi A, Williams BA, Mccue K, Schaeffer L, Wold B: **Mapping and quantifying mammalian transcriptomes by RNA-Seq.** *Nat Methods* 2008, **5:**621-628.
