# Supplementary material for: Interspecific and host-related gene expression patterns in nematode-trapping fungi
Source: BMC Genomics. 2014 Nov 11;15(1):968. doi: 10.1186/1471-2164-15-968 (PMC4237727; doi:10.1186/1471-2164-15-968)
Supplement: Supplementary file 2 — Additional file 2: Evaluations of procedures used for normalizing gene expression levels of UniRef50 clusters. (PDF 211 KB) [file 12864_2014_6662_MOESM2_ESM.pdf]

**Additional file 2. Evaluations of procedures used for normalizing gene expression levels of UniRef50 clusters**

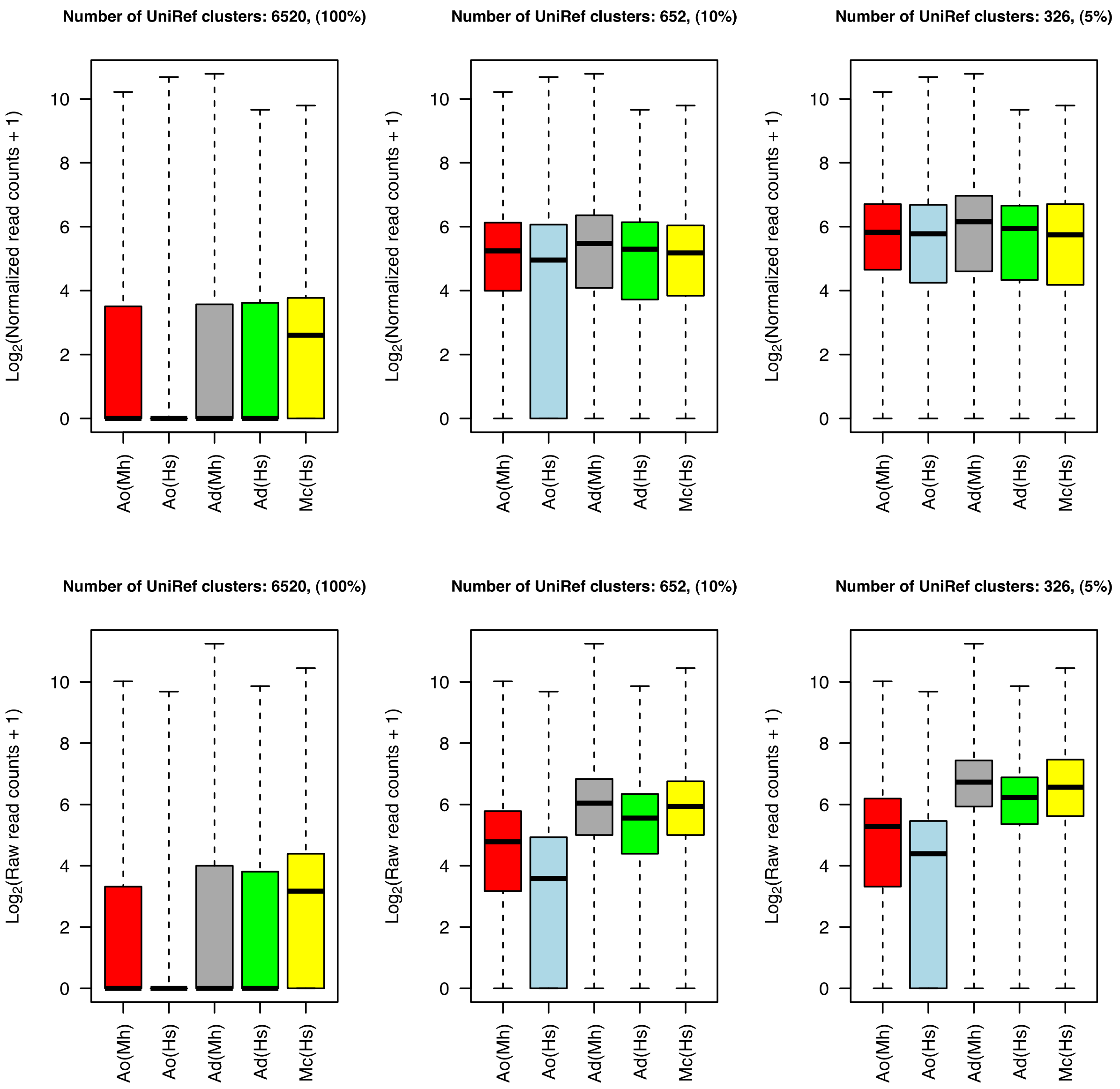


Isotig sequences were matched to UniRef50 clusters [1] and the reads were normalized using the DESeq method [2]. The line in the box represents the median of the read distribution whereas the bottom and the top of the box represent the 25$^{th}$ percentile and the 75$^{th}$ percentile, respectively. The minimum and maximum values are indicated by the ends of the vertical lines. Outliers are not shown in the box plots.

The box plots illustrate how the removal of UniRef50 clusters, with the lowest sum of read counts, affects the distribution of the expression values within each library. The percentages of UniRef50 clusters in different subsets (after the filtering) are shown within parentheses.

Normalized read counts are shown in the top row and raw read counts are shown in the bottom row.

Ao(Mh) denotes *A. oligospora* and *M. hapla*; Ao(Hs), *A. oligospora* and *H. schachtii*; Ad(Mh), *A. dactyloides* and *M. hapla*; Ad(Hs), *A. dactyloides* and *H. schachtii*; and Mc(Hs), *M. cionopagum* and *H. schachtii*.

## References


1. Suzek BE, Huang HZ, McGarvey P, Mazumder R, Wu CH: **UniRef: comprehensive and non-redundant UniProt reference clusters.** *Bioinformatics* 2007, **23:**1282-1288.

2. Anders S, Huber W: **Differential expression analysis for sequence count data.** *Genome Biol* 2010, **11:**R106.
