# Supplementary material for: Interspecific and host-related gene expression patterns in nematode-trapping fungi
Source: BMC Genomics. 2014 Nov 11;15(1):968. doi: 10.1186/1471-2164-15-968 (PMC4237727; doi:10.1186/1471-2164-15-968)
Supplement: Supplementary file 3 — Additional file 3: Summary of the annotation of the 500 most expressed transcripts. (PDF 94 KB) [file 12864_2014_6662_MOESM3_ESM.pdf]

Additional file 3. Summary of the annotation of the 500 most expressed transcripts[a]

	Ao(Mh)	Ao(Hs)	Ad(Mh)	Ad(Hs)	Mc(Hs)
**Number of isotigs**					
UniProt hit	471	473	431	442	444
Pfam domain[b]	388	373	354	368	370
Secreted	61	63	63	54	69
Orphans	39	38	64	48	46
KOG	369	379	365	364	358
EC numbers	27	28	12	12	14
UniRef50 clusters	436	445	367	393	370
**Number of Pfam domains**					
(Unique Pfam domains)	412	371	371	362	330
(Pfam counts)[c]	536	475	520	485	500

[a] The table shows the number of isotigs (i.e. transcripts) among the most highly expressed transcripts (Top 500 transcripts, c.f. Figure 2 in main text) that were annotated based on searches to the UniProt database [1], proteins from *M. haptotylum* [2], the Pfam protein family database [3], the presence of a predicted secretion signal [4], the KOG database [5] and the UniRef50 database [6]. Ao(Mh) denotes *A. oligospora* and *M. hapla*; Ao(Hs), *A. oligospora* and *H. schachtii*; Ad(Mh), *A. dactyloides* and *M. hapla*; Ad(Hs), *A. dactyloides* and *H. schachtii*; and Mc(Hs), *M. cionopagum* and *H. schachtii*. A complete list of the isotigs and their annotations are given in Additional file 4.

[b] The total number of transcripts that have Pfam domains among the Top 500 transcripts.

[c] The total number of Pfam domains among the Top500 transcripts. A domain occurring multiple times in a given isotig was counted once.

References

1. Apweiler R, Bairoch A, Wu CH, Barker WC, Boeckmann B, Ferro S, Gasteiger E, Huang HZ, Lopez R, Magrane M et al.: **UniProt: the Universal Protein knowledgebase.** *Nucleic Acids Res* 2004, **32:**115-119.

2. Meerupati T, Andersson KM, Friman E, Kumar D, Tunlid A, Ahrén D: **Genomic mechanisms accounting for the adaption to parasitism in nematode-trapping fungi.** *PLoS Genet* 2013, **9:**e1003909.

3. Finn RD, Mistry J, Tate J, Coggill P, Heger A, Pollington JE, Gavin OL, Gunasekaran P, Ceric G, Forslund K et al.: **The Pfam protein families database.** *Nucleic Acids Res* 2010, **38:**D211-D222.

4. Petersen TN, Brunak S, von Heijne G, Nielsen H: **SignalP 4.0: discriminating signal peptides from transmembrane regions.** *Nat Methods* 2011, **8:**785-786.

5. Tatusov RL, Fedorova ND, Jackson JD, Jacobs AR, Kiryutin B, Koonin EV, Krylov DM, Mazumder R, Mekhedov SL, Nikolskaya AN et al.: **The COG database: an updated version includes eukaryotes.** *BMC Bioinformatics* 2003, **4:**41.

6. Suzek BE, Huang HZ, McGarvey P, Mazumder R, Wu CH: **UniRef: comprehensive and non-redundant UniProt reference clusters.** *Bioinformatics* 2007, **23:**1282-1288.
