# Supplementary material for: Interspecific and host-related gene expression patterns in nematode-trapping fungi
Source: BMC Genomics. 2014 Nov 11;15(1):968. doi: 10.1186/1471-2164-15-968 (PMC4237727; doi:10.1186/1471-2164-15-968)
Supplement: Supplementary file 5 — Additional file 5: PCA analysis of highly expressed UniRef50 clusters. (PDF 94 KB) [file 12864_2014_6662_MOESM5_ESM.pdf]

Additional file 5. PCA analysis of highly expressed UniRef50 clusters

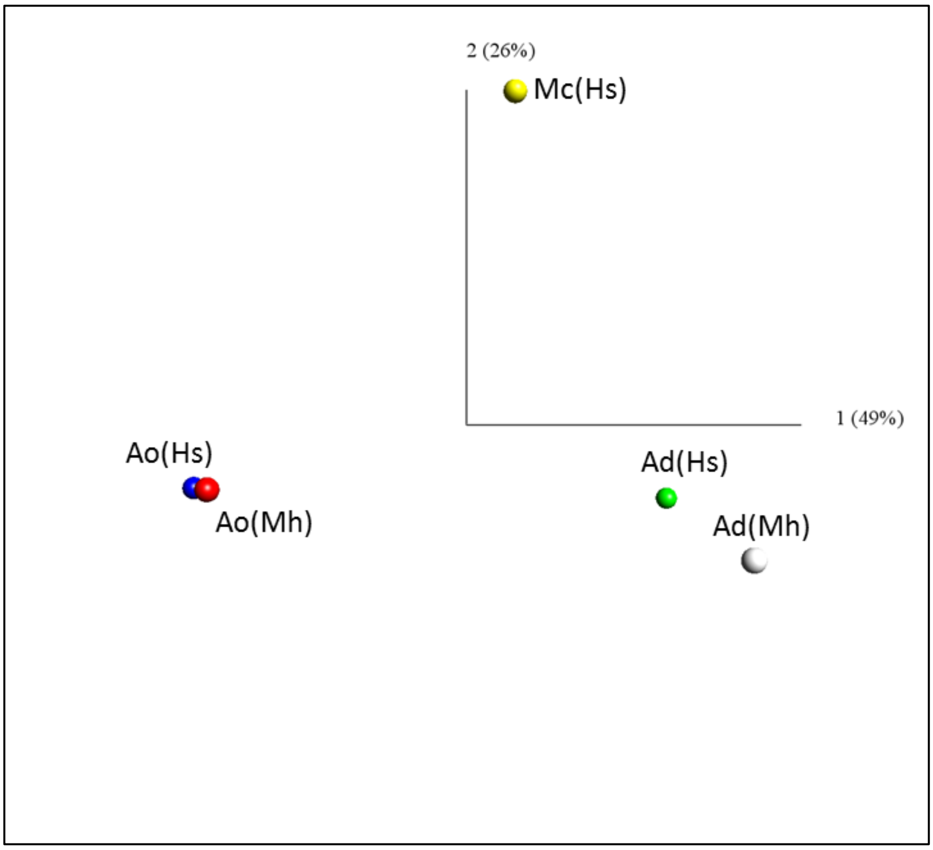

Each point in the PCA plot shows a cDNA library. Ao(Mh) denotes *A. oligospora* and *M. hapla*; Ao(Hs), *A. oligospora* and *H. schachtii*; Ad(Mh), *A. dactyloides* and *M. hapla*; Ad(Hs), *A. dactyloides* and *H. schachtii*; and Mc(Hs), *M. cionopagum* and *H. schachtii*. The PCA was performed on the expression levels (normalized, log_2-transformed read counts) of the 5% most highly expressed UniRef50 proteins represented by 326 unique IDs (c.f. Additional file 2).
