# Supplementary material for: Interspecific and host-related gene expression patterns in nematode-trapping fungi
Source: BMC Genomics. 2014 Nov 11;15(1):968. doi: 10.1186/1471-2164-15-968 (PMC4237727; doi:10.1186/1471-2164-15-968)
Supplement: Supplementary file 9 — Additional file 9: Virulence associated proteins highly expressed by all fungi. (PDF 165 KB) [file 12864_2014_6662_MOESM9_ESM.pdf]

Additional file 9. Virulence associated proteins highly expressed by all fungi[a]

PHI-base gene	Putative function	Ao(Mh)	Ao(Hs)	Ad(Mh)	Ad(Hs)	Mc(Hs)
TSA1	Antioxidant (Thiol peroxidase)	+	+	+	+	+
CNB1	Calcium binding protein (EF-hand motif)	+	+	+	+	+
CpkA	Cell signalling (Pkinase)	+	+	+	+	+
RAS1	Cell signalling (Ras)	+	+	+	+	+
RHO1	Cell signalling (Ras)	+	+	+	+	+
CLPT1[b]	Cell signalling (Ras)	+	+	+	+	+
MGG_02731	Cell signalling (Ras)	+		+	+	+
VAD1[b]	DEAD-box RNA helicase	+	+	+	+	+
PMR1	Membrane transport (Cation transport ATPase (P-type) family)	+		+		+
SIT4	Morphogenesis (Serine/threonine-protein phosphatase)	+	+	+	+	+
MNH6	Non-specific DNA-binding proteins (HMG box)	+	+	+	+	+
BcPIC5	Stress response (FKBP-type peptidyl-prolyl cis-trans isomerase)	+	+	+	+	+
CPA1	Stress response (Peptidylprolyl isomerase)	+	+	+	+	+
CaTUP1[b]	Transcription (Repressor Tup1)		+	+	+	+
HEX1	Woronin body major protein	+	+	+	+	+

[a] Shown is the presence (+) of transcripts displaying sequence similarity to proteins in the pathogen–host interaction protein database (PHI-base) [1]. The Top 500 transcripts in each library were analysed (Additional file 8) and shown are the PHI-base genes that were present in at least one library of each fungal species.

[b] PHI-base gene that at least ten gene models of *M. haptotylum* or *A. oligospora* showed sequence similarity to [2].

Reference List

1. Winnenburg R, Urban M, Beacham A, Baldwin TK, Holland S, Lindeberg M, Hansen H, Rawlings C, Hammond-Kosack KE, Kohler J: **PHI-base update: additions to the pathogen-host interaction database.** *Nucleic Acids Res* 2008, **36:**572-576.

2. Meerupati T, Andersson KM, Friman E, Kumar D, Tunlid A, Ahrén D: **Genomic mechanisms accounting for the adaption to parasitism in nematode-trapping fungi.** *PLoS Genet* 2013, **9:**e1003909.
