# Supplementary material for: Interspecific and host-related gene expression patterns in nematode-trapping fungi
Source: BMC Genomics. 2014 Nov 11;15(1):968. doi: 10.1186/1471-2164-15-968 (PMC4237727; doi:10.1186/1471-2164-15-968)
Supplement: Supplementary file 10 — Additional file 10: Variations in expression of virulence associated proteins. (PDF 68 KB) [file 12864_2014_6662_MOESM10_ESM.pdf]

Additional file 10. Variations in expression of virulence associated proteins[a]

PHI-base gene	Putative function	Ao(Mh)	Ao(Hs)	Ad(Mh)	Ad(Hs)	Mc(Hs)
PMT1	Adhesion/Morphogenesis (protein glycosylation gene)			+		+
CAT1	Antioxidant (Catalase)	+	+			+
SOD2	Antioxidant (Superoxide dismutase)	+	+			+
SOD1	Antioxidant (Superoxide dismutase)					+
SOD5	Antioxidant (Superoxide dismutase)		+			
SAP4	Aspartic protease			+		
SAP3	Aspartic protease					+
CNB1	Cell signalling (Ca^{2+} calmodulin-regulated phosphatase)				+	+
FGA1	Cell signalling (G protein)			+	+	
FGB1	Cell signalling (G protein)			+	+	
CPRGS-1	Cell signalling (G protein)			+	+	
MgRho3	Cell signalling (G protein)					+
BOS1	Cell signalling (Histidine kinase)			+		
bcplc1	Cell signalling (Phospholipase C)			+	+	
MPS1	Cell signalling (Pkinase)	+		+	+	
FMK1	Cell signalling (Pkinase)			+	+	+
PKAC1	Cell signalling (Pkinase)			+	+	+
Bmp3	Cell signalling (Pkinase)					+
CLK1	Cell signalling (Pkinase)			+		
UKC1	Cell signalling (Pkinase)					+
SNF1[b]	Cell signalling (Pkinase)			+		
RPK1	Cell signalling (Pkinase)					+
CZK3	Cell signalling (Pkinase)			+		
CaRAS1	Cell signalling (Ras)		+			
GEL2	Cell wall biogenesis (1,3-beta-glucanosyltransferase)	+				
CHS3	Cell wall biogenesis (Chitin synthase)			+		
WdCHS5	Cell wall biogenesis (Chitin synthase)		+			
Umchs6	Cell wall biogenesis (Chitin synthase)	+	+	+	+	
chsG	Cell wall biogenesis (Chitin synthase)		+	+	+	
VFGLU1	Cellulase		+			
MSP1	Cerato-platanin		+			+
RBT4[b]	Cysteine-rich secretory protein (Function unknown)	+	+			
ACE1	Effector (Avirulence Conferring Enzyme)	+	+			
hopI1	Effector, HSP70 interaction			+		
GAS1[b]	Gas 1	+	+			+
GAS1	Gas 1	+	+	+		
GAS2	Gas 2		+			
ABC3[b]	Membrane transport (ABC transporter)	+	+	+		
MLT1	Membrane transport (ABC transporter)		+			
PDE1	Membrane transport (ATPase)			+		
PLD1	Membrane transport/Cell signalling (Phospholipase D1)			+	+	
MGG_04556	Metabolism (Alcohol dehydrogenase 1)	+				+
CTB6	Metabolism, Cercosporin biosynthesis					+

CTB3	Metabolism, Cercosporin biosynthesis	+				
FAS2	Metabolism (Fatty acid synthase)			+	+	
TOXC	Metabolism (Fatty acid synthase)			+		
GNA1	Metabolism (Glucosamine 6-phosphate N-acetyltransferase)				+	+
MGG_04582	Metabolism (Glycoside hydrolase)	+	+			
MSY1	Metabolism (Methionine synthase)	+	+	+		
GzmetE	Metabolism (Methionine synthase)	+	+			
MET3	Metabolism (Methionine/cysteine biosynthetic)	+	+			
CaNAG5	Metabolism (N-Acetylglucosamine kinase)			+	+	
MGG_00056	Metabolism (Short-chain dehydrogenase/reductase)	+	+			
UGD1	Metabolism (UDP-glucose dehydrogenase)	+	+	+	+	
THIOL	Metabolism, (Thiolase)				+	
ODC	Metabolism, amino acids (Ornithine decarboxylase)	+	+		+	
MGG_00383	Metabolism (Methionine synthase gene)	+	+	+		
ILV2	Metabolism, isoleucine and valine synthesis (Acetolactate synthase)	+	+			
LYSF	Metabolism, lysine synthesis (Aconitase)			+		
SPE3-LYS9	Metabolism, lysine synthesis (Saccharopine dehydrogenase)	+				
CBL1	Metabolism, methionine synthesis (Cystathionine beta-lyase)	+	+			
TPS1	Metabolism, pentose phosphate pathway (Trehalose-6-phosphate synthase)	+	+		+	
MCSA	Metabolism, propanoate metabolism (Methylcitrate synthase)	+	+			
ADE2	Metabolism, purine biosynthesis (Phosphoribosylaminoimidazole carboxylase)		+			
HMR1	Metabolism, sterol and isoprenoid synthesis (Hydroxymethyl-glutaryl CoA reductase)					+
CDC11	Morphogenesis (GTP binding)			+	+	
CDC10	Morphogenesis (GTP binding)			+		
bcnoxR	NADPH oxidase				+	
PEX6	Peroxisome biogenesis		+			
PKS1	Polyketide synthase	+				
CHS7	Stress response (Chaperone)				+	
BCP1	Stress response (Peptidylprolyl isomerase)					+
GNO1	Stress response (S-nitrosoglutathione (GSNO) reductase)	+	+			
CPCA	Transcription (Cross-pathway control protein A transcription factor)					+
CON7	Transcription (Putative regulator)				+	
MST12	Transcription (Ste like transcription	+	+			+

	factor)				
CST1	Transcription (Ste12p like transcription factor)	+			+
FOW1	Transport (mitochondrial carrier gene)			+	
ClaSSD1	Unknown		+		+
ORP1	Unknown		+		
MGG_03530	Unknown				+
MGG_09263	Unknown				+

[a] Shown is the presence (+) of transcripts displaying sequence similarity to proteins in the pathogen–host interaction protein database (PHI-base) [1]. The Top 500 transcripts in each library were analysed (Additional file 8) and shown are the PHI-base genes that were present in one or two but not in all three fungal species.

[b] PHI-base gene that at least ten gene models of *M. haptotylum* or *A. oligospora* showed sequence similarity to [2].

Reference List

1. Winnenburg R, Urban M, Beacham A, Baldwin TK, Holland S, Lindeberg M, Hansen H, Rawlings C, Hammond-Kosack KE, Kohler J: **PHI-base update: additions to the pathogen-host interaction database.** *Nucleic Acids Res* 2008, **36:**572-576.

2. Meerupati T, Andersson KM, Friman E, Kumar D, Tunlid A, Ahrén D: **Genomic mechanisms accounting for the adaption to parasitism in nematode-trapping fungi.** *PLoS Genet* 2013, **9:**e1003909.
