# Supplementary material for: Interspecific and host-related gene expression patterns in nematode-trapping fungi
Source: BMC Genomics. 2014 Nov 11;15(1):968. doi: 10.1186/1471-2164-15-968 (PMC4237727; doi:10.1186/1471-2164-15-968)
Supplement: Supplementary file 11 — Additional file 11: Upregulated genes in A. oligospora during infection of M. hapla as compared with H. schachtii. (PDF 104 KB) [file 12864_2014_6662_MOESM11_ESM.pdf]

Additional file 11. Upregulated genes in *A. oligospora* during infection of *M. hapla* as compared with *H. schachtii*[a]

UniProt	Pfam	SignalP[b]	Orphan[c]	Ao(Mh) Read counts[d]	Ao(Hs) Read counts[d]	Fold Change
G1X4P0	His_Phos_2	Y	-	12.1	1.3	9.1
G1XTC9	Patched	Y	-	12.1	1.3	9.1
G1XF88	-	Y	-	20.4	2.6	7.7
G1XGH3	-	Y	-	9.8	1.3	7.4
G1XHE8	-	Y	Y	9.1	1.3	6.9
G1X1U4	-	Y	-	8.3	1.3	6.3
G1X8R3	But2	Y	-	40.8	6.6	6.2
G1XCM7	Glyco_hydro_35,BetaGal_dom2,BetaGal_dom3,BetaGal_dom4_5	Y	-	7.6	1.3	5.7
G1XET4	Peptidase_S41	Y	-	7.6	1.3	5.7
G1XGI5	-	Y	-	21.2	4.0	5.3
G1XPV3	-	Y	-	104.3	19.8	5.3
G1XJP0	-	Y	-	20.4	4.0	5.1
G1XLE5	PA,Peptidase_M28	Y	-	6.8	1.3	5.1
G1XAY3	Transp_cyt_pur	-	-	26.5	1.3	20.0
G1XRI4	-	-	-	38.6	2.6	14.6
G1X7I7	-	-	-	36.3	2.6	13.7
G1XQ65	CIAPIN1	-	-	13.6	1.3	10.3
G1XD10	-	-	-	13.6	1.3	10.3
G1XA82	Fungal_lectin	-	-	12.9	1.3	9.7
G1XAF6	GCV_T,GCV_T_C	-	-	12.9	1.3	9.7
G1X6N1	SH3_1,Peroxin-13_N	-	-	12.9	1.3	9.7
G1X6S6	UCH	-	-	12.9	1.3	9.7
G1XBS6	MFS_1	-	-	12.1	1.3	9.1
G1XMJ1	-	-	-	12.1	1.3	9.1
G1X6D0	zf-MYND	-	-	12.1	1.3	9.1
G1X2Y3	DEAD,Helicase_C,Sec63	-	-	10.6	1.3	8.0
G1XKI7	DENN,C1_2,dDENN,uDENN	-	-	10.6	1.3	8.0
G1XDB6	-	-	-	10.6	1.3	8.0
G1X5K9	-	-	-	19.7	2.6	7.4
G1XDD9	Pkinase	-	-	19.7	2.6	7.4
G1WYS8	A_deaminase	-	-	9.8	1.3	7.4
G1X2Y8	FGGY_N,FGGY_C	-	-	9.8	1.3	7.4
G1X0A1	-	-	-	9.8	1.3	7.4
G1XSL4	-	-	-	9.8	1.3	7.4

G1XK29	-	-	-	28.0	4.0	7.0
G1XCD5	Sugar_tr	-	-	27.2	4.0	6.9
G1X2W7	CBFD_NFYB_HMF	-	-	9.1	1.3	6.9
G1X1X5	-	-	-	9.1	1.3	6.9
G1X574	Phosphodiest,PigN	-	-	9.1	1.3	6.9
G1X6B8	Snf7	-	-	9.1	1.3	6.9
G1XMJ0	TIG,Ank_2	-	-	9.1	1.3	6.9
G1XV94	PTR2	-	-	24.9	4.0	6.3
G1X301	bZIP_1	-	-	8.3	1.3	6.3
G1XUI0	Cut12	-	-	8.3	1.3	6.3
G1X8D3	Glyco_hydro_31,Gal_ mutarotas_2	-	-	8.3	1.3	6.3
G1XI69	Myb_DNA-binding	-	-	8.3	1.3	6.3
G1XRE3	-	-	-	8.3	1.3	6.3
G1XIV0	p450	-	-	8.3	1.3	6.3
G1XP36	PLDc,PX,PLDc_2	-	-	8.3	1.3	6.3
G1X891	TPMT	-	-	8.3	1.3	6.3
G1XQV9	ADH_zinc_N,ADH_N	-	-	24.2	4.0	6.1
G1X4Y1	-	-	-	15.9	2.6	6.0
G1X583	-	-	-	15.1	2.6	5.7
G1XJL1	-	-	-	15.1	2.6	5.7
G1XJN8	-	-	-	15.1	2.6	5.7
G1XTH4	ApbA,ApbA_C	-	-	7.6	1.3	5.7
G1X9K7	APS_kinase	-	-	7.6	1.3	5.7
G1XDH6	Citrate_synt	-	-	7.6	1.3	5.7
G1XBW2	DEP,RhoGEF,CNH	-	-	7.6	1.3	5.7
G1XQ38	GDPD,SPX,Ank_2	-	-	7.6	1.3	5.7
G1XJI2	Glyco_hydro_15	-	-	7.6	1.3	5.7
G1X9B6	-	-	-	7.6	1.3	5.7
G1XE06	-	-	-	7.6	1.3	5.7
G1XES5	-	-	-	7.6	1.3	5.7
G1XK33	-	-	-	7.6	1.3	5.7
G1XKP1	-	-	-	7.6	1.3	5.7
G1XJ81	PDH,2-Hacid_dh_C	-	-	7.6	1.3	5.7
G1X7E2	Prenyltrans	-	-	7.6	1.3	5.7
G1XBA1	Prenyltransf	-	-	7.6	1.3	5.7
G1XTD9	Pyridox_ox_2	-	-	7.6	1.3	5.7
G1WZV4	SIR2	-	-	7.6	1.3	5.7
G1XRC1	Sugar_tr	-	-	7.6	1.3	5.7
G1X2K0	UBX,SEP	-	-	7.6	1.3	5.7
G1X4D1	WD40	-	-	7.6	1.3	5.7
G1X6E6	WD40	-	-	7.6	1.3	5.7
G1XFL4	ZZ	-	-	7.6	1.3	5.7
G1X302	-	-	-	14.4	2.6	5.4
G1XTM0	TPP_enzyme_M,TPP_e	-	-	21.2	4.0	5.3

	nzyme_C,TPP_enzyme_N					
G1WXZ2	DUF2823	-	-	49.1	9.3	5.3
G1X9I8	Aa_trans	-	-	6.8	1.3	5.1
G1XBU0	Aa_trans	-	-	6.8	1.3	5.1
G1XKI9	Aa_trans	-	-	6.8	1.3	5.1
G1X418	Cyclin	-	-	6.8	1.3	5.1
G1X6F7	DnaJ,DnaJ_CXXCXGXG,DnaJ_C	-	-	6.8	1.3	5.1
G1X6S2	Epimerase	-	-	6.8	1.3	5.1
G1WZR4	HAT,TPR_8	-	-	6.8	1.3	5.1
G1X4P3	HLH	-	-	6.8	1.3	5.1
G1XB73	LMBR1	-	-	6.8	1.3	5.1
G1WXS1	Mannosyl_trans	-	-	6.8	1.3	5.1
G1WXM2	-	-	-	6.8	1.3	5.1
G1WYE5	-	-	-	6.8	1.3	5.1
G1WZN6	-	-	-	6.8	1.3	5.1
G1X020	-	-	-	6.8	1.3	5.1
G1X225	-	-	-	6.8	1.3	5.1
G1X944	-	-	-	6.8	1.3	5.1
G1XB50	-	-	-	6.8	1.3	5.1
G1XRZ3	-	-	-	6.8	1.3	5.1
G1WXU7	Peptidase_M28	-	-	6.8	1.3	5.1
G1XKD2	Pkinase	-	-	6.8	1.3	5.1
G1XSE3	PNP_UDP_1	-	-	6.8	1.3	5.1
G1X8J5	RhoGAP	-	-	6.8	1.3	5.1
G1XBU9	SH3_1	-	-	6.8	1.3	5.1
G1X6Y1	zf-H2C2	-	-	6.8	1.3	5.1
G1X4E2	Zip	-	-	6.8	1.3	5.1
G1X528	Zn_clus,Fungal_trans	-	-	6.8	1.3	5.1

[a] Shown are 105 genes that were upregulated at least 5-fold in *M. hapla* (Ao(Mh)) as compared to *H. schachtii* (Ao(Hs)).Genes that were expressed (≥1 read) in both libraries and that had ≥5 read in any of the libraries were included in the analysis, in total 4,138 genes.

[b] Y denotes protein that has a predicted secretion signal.

[c] Y denotes protein that lack known homologs and do not contain any Pfam domains.

[d] Normalized read count using DESeq [1].

References

1. Anders S, Huber W: **Differential expression analysis for sequence count data.** *Genome Biol* 2010, **11:**R106.
