# Supplementary material for: Interspecific and host-related gene expression patterns in nematode-trapping fungi
Source: BMC Genomics. 2014 Nov 11;15(1):968. doi: 10.1186/1471-2164-15-968 (PMC4237727; doi:10.1186/1471-2164-15-968)
Supplement: Supplementary file 12 — Additional file 12: Upregulated genes in A. oligospora during infection of H. schachtii as compared with M. hapla. (PDF 85 KB) [file 12864_2014_6662_MOESM12_ESM.pdf]

Additional file 12. Upregulated genes in *A. oligospora* during infection of *H. schachtii* as compared with *M. hapla*[a]

UniProt	Pfam	SignalP[b]	Orphan[c]	Ao(Mh) Read counts[d]	Ao(Hs) Read counts[d]	Fold change
G1X7Q6	-	Y	Y	0.8	11.9	15.8
G1XQA9	-	Y	-	0.8	11.9	15.8
G1XU57	ABC_tran,ABC2_membrane	Y	-	0.8	11.9	15.8
G1X110	-	Y	Y	1.5	17.2	11.4
G1XR64	DUF3129	Y	-	1.5	15.9	10.5
G1XF27	WSC	Y	-	3.0	29.1	9.6
G1XEV7	Glyco_hydro_18,LysM	Y	-	0.8	6.6	8.8
G1XM84	Tyrosinase	Y	-	0.8	6.6	8.8
G1XC55	DUF3129	Y	-	2.3	14.6	6.4
G1X2P2	XPG_N,XPG_I	-	-	0.8	11.9	15.8
G1XI73	Peptidase_S8	-	-	0.8	10.6	14.0
G1XFT7	PNP_UDP_1	-	-	0.8	9.3	12.3
G1XGH0	AAA	-	-	0.8	9.3	12.3
G1XKL8	-	-	-	0.8	9.3	12.3
G1XPG7	-	-	-	0.8	9.3	12.3
G1XQD0	-	-	-	0.8	9.3	12.3
G1XUQ9	-	-	-	0.8	9.3	12.3
G1XP78	-	-	Y	0.8	7.9	10.5
G1X2W0	Swi3	-	-	0.8	7.9	10.5
G1X3U4	-	-	-	0.8	7.9	10.5
G1X4J9	G_glu_transpept	-	-	0.8	7.9	10.5
G1X5S8	-	-	-	0.8	7.9	10.5
G1XD25	-	-	-	0.8	7.9	10.5
G1XD93	-	-	-	0.8	7.9	10.5
G1XJ93	-	-	-	0.8	7.9	10.5
G1XJM0	-	-	-	0.8	7.9	10.5
G1X990	Ribosomal_L36e	-	-	6.0	56.9	9.4
G1XNI8	-	-	Y	0.8	6.6	8.8
G1X8J0	KTI12	-	-	0.8	6.6	8.8
G1XAH7	Prefoldin_2	-	-	0.8	6.6	8.8
G1XAR3	-	-	-	0.8	6.6	8.8
G1XC75	ABC_tran,ABC_membrane	-	-	0.8	6.6	8.8
G1XEI0	CorA	-	-	0.8	6.6	8.8
G1XG98	DDHD	-	-	0.8	6.6	8.8
G1XHG0	GatB_Yqey,GatB_N	-	-	0.8	6.6	8.8
G1XSM5	-	-	-	0.8	6.6	8.8
G1XH43	Ribosomal_L7Ae	-	-	1.5	11.9	7.9

G1WZX1	Mito_carr	-	-	1.5	10.6	7.0
G1XGL7	-	-	-	1.5	10.6	7.0
G1X111	-	-	-	1.5	9.3	6.1
G1X1V9	-	-	-	1.5	9.3	6.1
G1X3D8	-	-	-	1.5	9.3	6.1
G1X5Q2	-	-	-	1.5	9.3	6.1
G1XBK3	-	-	-	1.5	9.3	6.1
G1XUJ4	MFS_1	-	-	1.5	9.3	6.1
G1XFY8	MFS_1	-	-	6.0	35.7	5.9
G1X402	-	-	-	2.3	13.2	5.8
G1XIS6	-	-	-	3.0	17.2	5.7
G1XRN5	-	-	-	3.0	17.2	5.7
G1XHX4	-	-	Y	1.5	7.9	5.3
G1X6X7	-	-	-	1.5	7.9	5.3
G1XA48	DAGK_cat	-	-	1.5	7.9	5.3
G1XAV2	-	-	-	1.5	7.9	5.3
G1XD31	MMR_HSR1	-	-	1.5	7.9	5.3
G1XD67	NB-ARC	-	-	1.5	7.9	5.3
G1XPA7	TPR_8	-	-	1.5	7.9	5.3
G1XQ85	ALAD	-	-	1.5	7.9	5.3
G1XQ89	IF-2B	-	-	1.5	7.9	5.3
G1XTQ9	DNA_pol_E_B,Pol_alpha_B_N	-	-	1.5	7.9	5.3
G1XV20	Response_reg,HisKA,HATPase_c	-	-	1.5	7.9	5.3
G1WXN2	WD40	-	-	2.3	11.9	5.3
G1XSA8	PNP_UDP_1	-	-	2.3	11.9	5.3
G1XTV2	-	-	-	2.3	11.9	5.3
G1XUD3	MBOAT	-	-	2.3	11.9	5.3
G1XGE3	GTP_EFTU	-	-	3.0	15.9	5.3

[a] Shown are 65 genes that were upregulated at least 5-fold in *H. schachtii* (Ao(Hs)) as compared to *M. hapla* (Ao(Mh)). Genes that were expressed (≥1 read) in both libraries and that had ≥5 read in any of the libraries were included in the analysis, in total 4,138 genes.

[b] Y denotes protein that has a predicted secretion signal.

[c] Y denotes protein that lack known homologs and do not contain any Pfam domains.

[d] Normalized read count using DESeq [1].

References

1. Anders S, Huber W: **Differential expression analysis for sequence count data.** *Genome Biol* 2010, **11:**R106.
